# Glymphatic distribution of CSF-derived apoE into brain is isoform specific and suppressed during sleep deprivation

**DOI:** 10.1186/s13024-016-0138-8

**Published:** 2016-12-08

**Authors:** Thiyagaragan M. Achariyar, Baoman Li, Weiguo Peng, Philip B. Verghese, Yang Shi, Evan McConnell, Abdellatif Benraiss, Tristan Kasper, Wei Song, Takahiro Takana, David M. Holtzman, Maiken Nedergaard, Rashid Deane

**Affiliations:** 1 Center for Translational Neuromedicine, Division of Glial Disease and Therapeutics, Department of Neurosurgery, University of Rochester Medical Center, University of Rochester, Rochester, NY 14642 USA; 2 Department of Neurology, Hope Center for Neurological Disorders, and the Charles F. and Joanne Knight Alzheimer’s Disease Research Center, Washington University School of Medicine, St Louis, MO 63110 USA; 3 Center for Translational Neuromedicine, Division of Cell and Gene Therapy, University of Rochester Medical Center, Rochester, NY 14642 USA; 4 Laboratory of Brain Metabolic Diseases, Institute of Metabolic Disease Research and Drug Development, China Medical University, Shenyang, China

**Keywords:** Glymphatic pathways, AQP4, Alzheimer’s disease, Lymphatic system, Brain clearance, Sleep/wake

## Abstract

**Background:**

Apolipoprotein E (apoE) is a major carrier of cholesterol and essential for synaptic plasticity. In brain, it’s expressed by many cells but highly expressed by the choroid plexus and the predominant apolipoprotein in cerebrospinal fluid (CSF). The role of apoE in the CSF is unclear. Recently, the glymphatic system was described as a clearance system whereby CSF and ISF (interstitial fluid) is exchanged via the peri-arterial space and convective flow of ISF clearance is mediated by aquaporin 4 (AQP4), a water channel. We reasoned that this system also serves to distribute essential molecules in CSF into brain. The aim was to establish whether apoE in CSF, secreted by the choroid plexus, is distributed into brain, and whether this distribution pattern was altered by sleep deprivation.

**Methods:**

We used fluorescently labeled lipidated apoE isoforms, lenti-apoE3 delivered to the choroid plexus, immunohistochemistry to map apoE brain distribution, immunolabeled cells and proteins in brain, Western blot analysis and ELISA to determine apoE levels and radiolabeled molecules to quantify CSF inflow into brain and brain clearance in mice. Data were statistically analyzed using ANOVA or Student’s t- test.

**Results:**

We show that the glymphatic fluid transporting system contributes to the delivery of choroid plexus/CSF-derived human apoE to neurons. CSF-delivered human apoE entered brain via the perivascular space of penetrating arteries and flows radially around arteries, but not veins, in an isoform specific manner (apoE2 > apoE3 > apoE4). Flow of apoE around arteries was facilitated by AQP4, a characteristic feature of the glymphatic system. ApoE3, delivered by lentivirus to the choroid plexus and ependymal layer but not to the parenchymal cells, was present in the CSF, penetrating arteries and neurons. The inflow of CSF, which contains apoE, into brain and its clearance from the interstitium were severely suppressed by sleep deprivation compared to the sleep state.

**Conclusions:**

Thus, choroid plexus/CSF provides an additional source of apoE and the glymphatic fluid transporting system delivers it to brain via the periarterial space. By implication, failure in this essential physiological role of the glymphatic fluid flow and ISF clearance may also contribute to apoE isoform-specific disorders in the long term.

**Electronic supplementary material:**

The online version of this article (doi:10.1186/s13024-016-0138-8) contains supplementary material, which is available to authorized users.

## Background

Apolipoprotein E (apoE), a 34 kDa protein, regulates the transport and metabolism of cholesterol in the periphery and central nervous system (CNS) [1–3]. In humans, there are three major apoE isoforms that differ in one or two amino acids; apoE2 (cysteine112, cysteine158), apoE3 (cysteine112, arginine158) and apoE4 (arginine112, arginine158). In European Americans, the prevalence of the ε2, ε3 and ε4 alleles is 7, 78 and 15%, respectively [4]. The most profound pathological consequence attributed to apoE polymorphism is the strong association of the ε4 allele with neurodegeneration [5].

The CNS relies primarily on de novo synthesis of apoE, since the blood–brain barrier (BBB) restricts the transport of apoE and cholesterol into and out of brain [6–8]. Mature neurons have a high demand for cholesterol and while they can synthesize it, under physiological conditions additional supplement in the form of apoE-associated cholesterol is used [9]. This task has been outsourced to non-neuronal cells, the glial cells, and in particular astrocytes, considered the main producer of brain apoE [10–16]. However, the choroid plexus highly expresses apoE [17], and apoE is one of the major apolipoprotein in the cerebrospinal fluid (CSF) [3, 17–24]. The role of apoE in the CSF is unclear.

In the periphery, apoE is produced mainly by the liver and distributed body-wide, except to the brain, via the blood and lymphatic systems [25]. To our knowledge, it has not been considered whether a brain-wide mechanism for the distribution of CSF apoE exists. Recently, the ‘glymphatic system’, a fluid transporting pathway that is functionally analogous to the peripheral lymphatic system, was shown to be fundamental for the bulk flow/convective flow of brain interstitial fluid (ISF) and its downstream clearance. This system consists of CSF inflow via the perivascular space of penetrating arteries, CSF/ISF exchange and astrocytic aquaporin 4 (AQP4)-mediated bulk flow of ISF through the parenchyma [26–30]. We reasoned that this system is not only waste removal pathways but should also distribute molecules in the CSF, such as choroid plexus-derived apoE, to brain. We tested the hypothesis that the glymphatic fluid transport also serves as delivery and distribution pathways for choroid plexus-derived apoE to brain. ApoE was used since its brain derived, confined within the CNS, a known risk factor for neurodegeneration and a potential therapy for Alzheimer’s disease (AD). The aims of these studies were to establish whether apoE in the CSF and apoE secreted by the choroid plexus into CSF were distributed into brain via the peri-arterial space, and whether this distribution pattern was altered by sleep deprivation. The study was necessary to determine whether apoE in CSF can enter brain and taken up by parenchymal cells, such as neurons, since astrocytes are believed to be the major source of brain apoE. It is important to the field as it provides an alternative means to target the brain by circumventing the BBB.

Our analysis shows that the glymphatic fluid transport plays an important role in the macroscopic delivery of apoE, produced by the choroid plexus, to brain. CSF-derived apoE is rapidly delivered into brain in a radial pattern around the penetrating arterial vessels, but not veins, in an isoform specific manner (apoE2 > apoE3 > apoE4) and associated with neurons. ApoE parenchymal distribution was facilitated by AQP4. In addition, sleep deprivation suppresses glymphatic CSF-derived apoE distribution into brain and its clearance. Thus, speculating, failure of the glymphatic fluid transport may contribute to apoE related disorders, in the long-term.

## Methods

### Mice

Male C57BL/6 J and NG2-DsRed (Tg(Cspg4-Ds Red.T1)1Akik/J) mice, which are on C57BL/6 J genetic background, were purchased from Jackson Laboratory (Bar Harbor, ME). In mice older than 6–8 weeks, oligodendrocyte progenitor cells rarely express Ds-Red, which is restricted to peri-vascular smooth muscle cells and pericytes [26, 31]. In these mice, arteries and arterioles are labeled with DsRed, whereas veins are DsRed negative [26]. The AQP4 ko (*Aqp4* ^*−/−*^ *)* mice were obtained from Dr Nagelhus and generated as described [32]. While there was no significant sex differences in glymphatic fluid transport of apoE in young mice, males were used for consistency. The mice were housed in the vivarium facilities at the University of Rochester, School of Medicine and Dentistry. All animal studies were performed according to the NIH guidelines using protocols approved by the University of Rochester Committee on Animal Resources. Mice were housed at a maximum of five per cage, as permitted, depending on the experiment, and maintained on a 12:12 light/dark schedule (6 AM: 6 PM) with food and water ad libitum. Mice were anesthetized with a mixture of ketamine (100 mg/kg) and xylazine (10 mg/kg) by intraperitoneal injection (IP).

### Materials

The lentivirus (pTANK-CMVie-LckEGFP-WPRE) expressing membrane-bound enhanced green fluorescent protein (EGFP) was designed to carry, in the 5' to 3' direction, a central polypurine tract (cPPT) element, a cytomegalovirus (CMV) immediate early promoter, membrane-bounded EGFP, expressed in tandem with a Woodchuck Hepatitis Virus Posttranscriptional Regulatory Element (WPRE). Virus particles (called lenti-EGFP) pseudo typed with vesicular stomatitis virus G glycoprotein were produced, concentrated by ultracentrifugation, and titrated on 293HEK cells (1.0 x10^12^ colony forming unit/ml) [33, 34]. Lentivirus-apoE3 (OHS 5899–202616165; Precision LentiORF.APOE3 with stop codon) was obtained from GE Dharmacon, Inc. (Lafayette, CO, USA). The apoE3 was cloned into LentiORD plasmid under the control of the CMV promoter. The expression cassette contains CMV, apoE3, IRES, EGFP and WPRE. We referred to this apoE3 gene delivery as lenti-apoE3 to compare with the lenti-EGFP particle. The internal ribosome entry site (IRES) provides for bicistronic expression, but the expression of EGFP is low compared to that of apoE3 in this cassette. Thus, to get a good signal of EGFP expression that is similar to that of apoE3 we used a mixture of both lenti-EGFP and lenti-apoE3 to provide a more rigorous test of the transduced cells (EGFP) in the presence of released apoE3 (detected by immunolabeling of the human apoE) in some experiments where stated. Reconstituted human lipidated-apoE2, −apoE3 and -apoE4 were obtained from Dr. David Holtzman. The particle size (10–17 nm) and disc shape were similar to the range of HDL-like particles derived from astrocytes or isolated from human CSF [12, 14, 15, 35, 36], and also similar to the reconstituted apoE [35]. The size and shape of lipoproteins in CSF are heterogeneous and may be species dependent [3, 37]. We used human apoE, the most abundant apolipoprotein in CSF, and our analysis only detects apoE. The apoE particles were purified by gel filtration-FPLC (Fast Protein Liquid Chromatography) to minimize the loss of proteins and antibody affinity column, and the particle size determined by non-denaturing gel electrophoresis followed Western blot analysis [12, 35]. In addition, the particle size of each of the three apoE isoform was similar [35]. ISF clearance of apoE is not affected by its particle size [6]. The particle size is within the size of the gaps (~20 nm) between astrocytic end feet [38, 39]. FITC-tagged apoE was prepared following the manufacturer’s instructions (FITC Protein labeling Kit (F-6434, Life Technologies, Carlsbad, CA, USA)*.* ApoE labeled with Alexa Fluor 647 was prepared following the manufacturer’s instructions (Alexa Fluor 647, Microscale Protein Labeling Kit (A30009), Molecular Probes, Inc., Eugene, OR, USA). Lectin was obtained from Vector Laboratory (Lycopersicon esculentum (tomato); Burlingame CA, USA).

### Transduction

Lentivirus carrying only EGFP or human apoE3 was stereotactically injected into the right lateral ventricle (co-ordinates: anteroposterior, −0.4 mm; mediolateral, +1.0 mm and dorsoventral, −2.3 mm), using a pulled glass pipette connected to a Hamilton syringe that is driven by a microinjector pump controller (Micro 4, World Precision Instruments, Inc., Sarasota, FL), at 1 μL/min for 3 min. At the end of the injection, the pipette was left at the injection site for 10 min to prevent reflux of the injectate along the injection track. It was removed slowly over 2 min. After 1 to 8 weeks post-transduction mice were re-anesthetized and perfusion fixed with 4% paraformaldehyde (PFA). Brains were sectioned horizontal at 100 μm thickness using a vibratome (Vibratome Series 1000). Sections were observed for EGFP expression, and processed for immunolabeling of various proteins, including human apoE.

### Immunohistochemistry

Immunohistochemistry was performed as reported [40, 41]. Mice were transcardially perfused with ice-cold phosphate buffered saline (PBS, pH 7.4, Sigma-Aldrich, St. Louis,MO,USA) followed by 4% PFA (Sigma-Aldrich). Free floating brain sections (100 μm; horizontal or coronal) were immunolabeled for various specific markers, such as neurons (NeuN) and astrocytes (GFAP). The tissue was blocked with 7% donkey serum for 1 h and incubated with the primary antibodies overnight. The primary antibodies were rabbit anti-human specific anti-apoE (1:200, Abcam Inc., Cambridge, MA; ab52607), mouse anti-aquaporin 1 (1:200, Abcam. Ab9566), mouse anti-GFAP (1:500, Sigma, G3893), chicken anti-NeuN (1:500, Abcam, ab134014), chicken anti-GFP antibody (Novus Biological,Littleton, CO,USA. NB100-1614). Then, Alexa Fluor-conjugated secondary antibodies were added and incubated for 2 h at room temperature (Life Technologies, 1:500). DAPI (Sigma, 1:2000) was used to identify cell nuclei. Immunofluorescence was visualized using a Bio-Rad MRC500 confocal scanning head attached to an inverted microscope (IX81, Olympus, Tokyo, Japan) controlled by Olympus Fluoview 500 software. For analysis of apoE intensities immunohistochemistry was performed on 100 μm vibratome-cut brain slices. Neurons were identified by NeuN labeling, astrocytes by GFAP expression and blood vessels by lectin staining. Arteries were identified by the red NG2-DsRed expression. NIH ImageJ software (1.47v) was used to measure the intensity of apoE immunoreactivity. Background intensities (5 per image) were measured in the parenchyma of the same field and subtracted from apoE intensities. The intensity of the immunolabeled apoE on the vessel wall and around (a 5 μm band around the vessel) arterial vessels or veins were quantified in the brain regions of three mice. For co-localization of apoE and neurons, a total of 30 neurons were quantified per brain and the average determined. The person analyzing the images and data was blinded to the experimental design*.*

### Intracisternal injections

Mice were anesthetized, fixed to a stereotactic frame and the cisterna magna exposed and cannulated using a 30G needle [26, 42]. Fluorescent tracers (5 μL aCSF containing apoE (0.5%) and cascade blue tagged dextran (10 kDa, 1%) as the reference inert molecule that is not significantly taken up by cells, transported across the BBB or degraded) were injected at 1 μl/min using a Hamilton syringe connected to a Micro Syringe pump controller (Micro 4; World Precision Instruments, Inc.). The same amount of apoE was used for each isoform for comparison. We used a small volume and a slow rate of injection (5 μL at 1 μL/min), that does not significantly increase the intracranial pressure [28], thereby minimizing CSF reflux into the cerebral ventricles from the cisterna magna [26]. ApoE4 and dextran (10 kDa) were used to characterize the technique since, in contrast to the dextran, apoE4 is retained within the brain to a greater extent compared to the other isoforms [6]. These tracers should provide a better resolution of CSF flow around arterial vessels. To quantify the glymphatic fluid inflow into brain, ^125^I-apoE, at a low concentration (10 nM, due to the greater resolution of radioactivity analysis), and ^14^C-inulin (6 kDa, 1.0 μCi, an inert molecule) were intracisternally injected at 1 μL/min for 5 min and after 30 min the brain removed and prepared for radioactivity analysis (see below).

### Lectin administration

Lectin was administered during the cardio-perfusion stage of the experiment, i.e., intravascularly. The mice were cardio-perfused with cold PBS, containing lectin (0.02 mg/ml), at 2 ml/min for 10 min. This was then followed by perfusion with PFA (4% in PBS), as described above.

### In vivo 2-Photon imaging

In anesthetized mice, a craniotomy (3 mm in diameter) was made over the sensory motor cortex. The dura was left intact and the craniotomy was covered with aCSF and sealed with a glass coverslip. To visualize the vasculature, 0.1 ml BBB impermeable Texas Red-dextran 70 (MW 70kD; 1% in saline, Invitrogen) was injected intravenously immediately before imaging. A Mai Tai laser (SpectraPhysics) attached to a confocal scanning system (Fluoview 300, Olympus) and an upright microscope (IX51W, Olympus) was used for in vivo imaging as described [26, 27]. A 20X (0.9NA) water immersion lens was used to image the cortex, from the surface to a depth of ~150 μm at every 5 μm z-steps. We used 870 nm as excitation wavelength and emission was collected at 575-645 nm. The cerebral vasculature was imaged at a resolution of 512 × 512px. FITC-apoE3 was administered intracisternally at 1 μL/min for 5 min and images acquired at 15 min after the injection.

### Sleep deprivation

Mice were sleep deprived from 6 to 9 AM and used immediately for the experiment at zeitgeber time 3 (ZT3). Sleep deprivation was maintained by using an enriched environment, containing novel objects and nesting material, to facilitate spontaneous exploration by the mice, as reported [43]. Mice were continuously observed to ensure that they were indeed awake and exploring the objects. Mice that were not exploring the objects were lightly prodded or gently handled to encourage their continuous activity. This method was chosen to minimize stress to the mice [43]. Food and water were freely available.

### Brain clearance

Earlier, it was shown that iodination (^125^I-) of lipidated apoE did not affect its clearance from brain ISF when compared to untagged lipidated apoE [44]. There are differenced in clearance rate between lipid-poor and lipidated apoE [6, 44]. Thus, if there were changes in the apoE lipidated state with iodination then it should have altered its clearance rate. To evaluate solute clearance from the brain, radio-labeled tracers (^125^I-apoE3 (prepared as we reported [6]) and ^14^C-inulin (6 kDa, PerkinElmer) were injected stereotaxtically into the left frontal cortex, as we recently reported [27]. Briefly, a stainless steel guide cannula (Plastic One) was implanted stereotaxically into the left frontal cortex of anesthetized mice (2% isoflurane) with the coordinates of the cannula tip at 0.7 mm anterior and 3.0 mm lateral to the bregma, and 1.0 mm below the surface of the brain. Animals were allowed to recover after surgery and the experiments performed 18–24 h after the guide tube cannulation, as reported [45]. Experiments during sleep state and sleep deprivation were performed between ZT3 and ZT4. In each mouse, a small volume of mock CSF (0.5 μL), containing ^125^I-apoE (10 nM) and ^14^C-inulin (1.0 μCi), was simultaneously injected (33 GA cannula, Plastic One) into the brain ISF over 5 min. At the end of the experiments (90 min) the brain was removed and prepared for radioactivity analysis. Samples were first counted for gamma radioactivity (^125^I-apoE) using Wallac 1471 Wizard Automatic Gamma Counter) and counts corrected for TCA-precipitability, as reported [45]. Then all samples were solubilized in 0.5 ml tissue solvable (Perkin Elmer) overnight followed by the addition of 5 ml of scintillation cocktail (Packard Ultima Gold). Samples were then analyzed in a liquid scintillation counter for ^14^C- radioactivity (LS6500 Multi-purpose Scintillation Counter (Beckman Coulter, GA, USA). *Calculations:* The percentage of radioactivity remaining in the brain after microinjection was determined as % recovery in brain = 100 x (N_b_/N_i_) (eq. 1), where, N_b_ is the radioactivity remaining in the brain at the end of the experiment and N_i_ is the radioactivity injected into the brain ISF. Total clearance was determined as 100-%recovery. ^14^C-inulin was used as an inert polar molecule which is neither transported across the BBB nor significantly retained by brain cells; its clearance provides a measure of the ISF bulk flow/convective flow. To confirm that FITC labeling apoE do not affect apoE clearance, we microinjected FITC-apoE3 or ^125^I-apoE3 intracortically, as above, and after 90 min the apoE3 levels remaining in brain were determined and expressed as a percentage of the injected dose that was cleared from brain, as reported [26, 46]. Levels of FITC-apoE3 were determined fluorometrically, as reported [47] and ^125^I-apoE3 analyzed as we reported [6].

### Flow (Diffusion) radius

A custom ImageJ plugin program was written to analyze apoE distribution radius as a function of distance from the vessel wall. In these studies, horizontal brain sections (100 μm each) from the cortex were used as there were no regional differences in apoE uptake from CSF. Arteries were identified by red NG2-DsRed (smooth muscles) expression in the NG2 DsRed reporter mice, veins were NG2 DsRed negative and the vasculature was labeled with lectin. The vessel diameter was calculated from the brain photomicrographs containing lectin immunofluorescence. The digital images of photomicrographs were used for the image analysis using imageJ software. The plugin plotted the intensity value of the immunofluorescence with respect to distance when a line was drawn across the blood vessel identified by the lectin immunofluorescence. Based on the change in the intensity value the blood vessel diameter was calculated. The cortical brain region was used since penetrating arteries were analyzed and there were no regional differences. All arterial vessels were analyzed in each brain section by a person blinded to the experimental design. Three horizontal brain sections (sensory-motor cortex) were used per mouse brain and there were 5 to 9 mice per group.

### Cell culture

Primary cultures of mouse astrocytes and choroid plexus epithelial cells were performed, as reported [40, 48]. In brief, the choroid plexus from the lateral ventricles and 4^th^ ventricle were removed from 15 C57BL6 male mice (4–8 weeks old), digested in 0.4% pronase in Hank’s balanced salt solution (HBSS) and cultured in Dulbecco’s modified Eagle’s medium (DMEM) containing fetal bovine serum (FBS), epidermal growth factor (EGF) and antibiotics (penicillin/streptomycin) on collagen coated wells. The purity of the choroid plexus epithelial cells was typically >99%, as determined from AQP1 immunolabeling and insignificant levels of astrocytes (GFAP-positive cells), and neurons (NeuN-positive cells). For cultured astrocytes, neocortical astrocytes were prepared from P1 to P4 C57BL/6 mouse pups, as previously described [40]. Briefly, cerebral cortices were separated and meninges were removed. The cortical tissue was dissected and washed three times in HBSS without Ca^2+^. After trituration and filtering through the 70-μm nylon mesh, the cells were centrifuged at 200 g for 10 min. The pellet was dissociated to single cell suspension in DMEM/F12, containing 1% Penicillin/Streptomycin and 10% FBS, and plated in T25 culture flasks. Typically, cultures with more than 99% of GFAP-positive cells were used in this study. All cells were grown at 37 °C in a humidified incubator (5% CO_2_/98% humidity). Medium was changed after 24 h and every 3 days thereafter. Conditioned media from the near confluent cells were collected, complete protease inhibitors (Roche Applied Sciences) added, centrifuged at 2000 g for 10 min to remove cellular debris and stored until required for analysis. Cells were then washed with PBS, harvested, pelleted and sonicated in lysis buffer.

### Brain extraction

Frozen cerebral cortex was sonicated in eight times volume of ice-cold RIPA lysis buffer, containing complete protease inhibitor (Roche Applied Sciences) and 30 mM β-mercaptoethanol (BME), and centrifuged at 10,000 g for 15 min in a microcentrifuge at 4 °C (Eppendorf). The supernatant was used to determine apoE levels using Western blot analysis. The same amount of total protein was loaded into each well. Protein levels were determined by using a BCA protein assay kit (ThermoFisher, Scientific).

### Western blot analysis

ApoE3 levels were determined by western blot analysis. Ice-cold CSF (10 μL) and standards (apoE3, 0.1 mg/ml) were diluted in an equal volume of Laemmli sample buffer (BioRad Laboratories, Hercules, CA; 161-0737) supplemented with 200 mM dithiothreitol (DTT; Sigma). Equal volumes (20 μL) of CSF and apoE3 standards were added to the sample wells of 4–15% Precast Gels (Mini-Protein TGX, BioRad, 456-0783) for electrophoresis under reducing conditions followed by transfer to a nitrocellulose membrane (BioRad, 162-0115) and detection using human specific anti-apoE antibody (Abcam, Ab52607; 1;1000) and a secondary antibody (goat anti-rabbit IgG-HRP, Santa Cruz sc2030; 1:10,000). For mouse apoE levels in brain a mouse specific anti-apoE with extremely low recognition of human apoE [49] was used (1:500; Ab20874, Abcam). Mouse apoE in the conditioned media was detected with an antibody (Calbiochem; Cat # 178479). Blots were developed using enhanced chemiluminescence (SuperSignal, 34077, Pierce, Rockford, IL). Levels of apoE3 in CSF were determined from the linear part of the standard curve that was generated from the intensities of Western blots of known amounts of apoE3. The relative abundance of human and mouse apoE in brain were determined with reference to β-actin. Conditioned media from the primary cultures of choroid epithelial cells and astrocytes were concentrated by ultrafiltration using a 10 kDa cut-off filter (Amicon Ultra Centrifuge Filters, 10 k NMWL, Merck Millipore, Cork, Ireland). The wells were loaded with equal volume of the concentrated conditioned media, and un-saturated intensities standardized to the protein concentration of the cultured cells.

### ApoE ELISA

Human apoE3 levels were determined using a human apoE specific ELISA kit (Abcam, Ab 108813) by following the manufacturer’s instructions. Samples of CSF from controls (Lenti-EGFP) were used to confirm human specificity. This signal was subtracted from the signal obtained in the CSF collected from the lenti-apoE3 transduced mice. Levels of apoE3 were determined from a standard curve.

### Statistical analysis

Data were analyzed using either Student’s *t* test when comparing two groups or analysis of variance (ANOVA) followed by post hoc Tukey test when comparing more than two groups. The differences were considered to be significant at *p* < 0.05. All values were expressed as mean ± SEM.

## Results

**Fig. 1:**
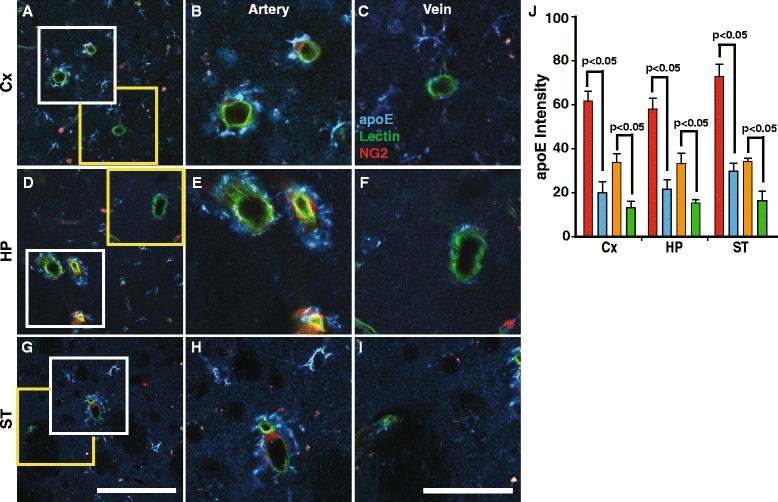
Greater levels of arterial endogenous mouse apoE than that on veins. Representative images of arteries and veins in brain regions (**a**, **d** and **g** at low magnification. Scale bar (100 μm). **b** cortical arteries with NG2-DsRed positive smooth muscle cells immunostained for apoE (*blue*) and (**c**) cortical vein lacking smooth muscle cells and with little apoE. Scale bar (50 μm). Similar pattern of apoE immunolabeling on arteries (**e** and **h**) and veins (**f** and **i**) in the hippocampus (**d**-**f**) and striatum (**g**-**i**). *Blue*, apoE; *red*, NG2-dsRed; *green*, Lectin. **j** Quantification of the apoE intensities on the vessel wall and around (a 5 μm circle around the vessel) arteries and veins in the different brain regions (cortex (Cx), hippocampus (HP) and striatum (ST)). Arterial wall (*red column*), peri-arterial (*orange*), vein (*blue*), peri-venous (*green*). White box (arterial vessels). Yellow box (venous vessels) Values are mean ± SEM. *N* = 4 mice per group

While apoE is expressed in many types of brain cells [17], astrocytic endfeet surrounding the large blood vessels displays high levels of apoE immunolabeling [50, 51], but it is unclear whether these vessels are arteries or veins. To assess whether apoE levels differ in the astrocytes surrounding arteries and veins, we used NG2-DsRed reporter mice in which arterial vessels (vascular smooth muscle cells) and pericytes, but not veins, are labeled with DsRed [26, 31]. Higher endogenous apoE immunolabeling around large arteries and arterioles than that around large veins in several regions (cortex, hippocampus and striatum) was consistently observed (Fig. 1a-i). While there were no regional differences, quantitative immunohistochemical analysis of apoE showed that the intensity on the vessel wall and around arterial vessels was about 2.3-3.0-fold and 2.1-2.5-fold greater compared to that on the walls and around veins, respectively (Fig. 1j). Since this distribution pattern may reflect a greater density of astrocyte on the arterial vessel walls compared to that of veins, astrocytes were immunolabeled with glial fibrillary acidic protein (GFAP), an astrocytic marker. The number of GFAP-positive astrocytic processes on arterial vessels that were labeled with endogenous apoE was greater than that on veins (Additional file 1: Figure S1A-G), as reported [52], which confirms the data shown in Fig. 1. Thus, this distinct distribution pattern may reflect higher levels of astrocytic-derived apoE around arterial vessels, or alternatively, that CSF apoE enters along the peri-arterial space and is taken up primarily by astrocytes surrounding larger arteries and arterioles. If so, this spatial distribution of endogenous apoE suggests the existence of a polarized (arteries to veins) transport system that facilitates its distribution within the ISF.

**Fig. 2:**
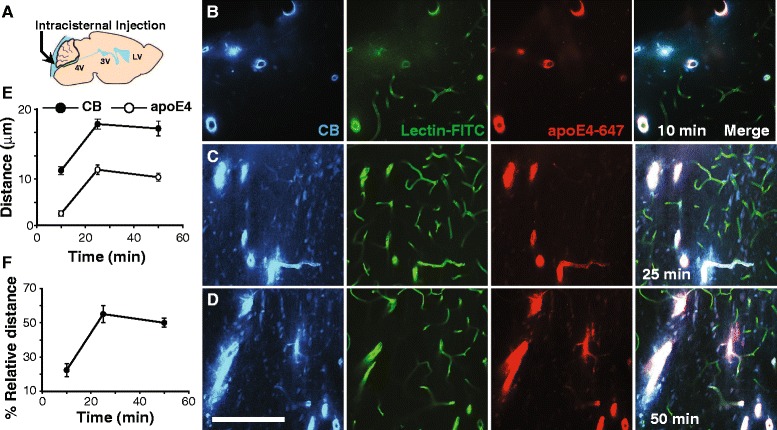
Distribution of CSF apoE4 around arteries but not veins (**a**) Schematic diagram showing the intracisternal injection site. **b-d** Representative images of apoE4-647 (*red*) and dextran-cascade blue (CB) radial distribution at 10, 25 and 50 min post-intracisternal injection. Scale bar = 100 μm. **e** Distribution distance of apoE4 and CB at different post-injection times. **f** ApoE4 distribution distance standardized as a percentage of that of CB. Values are mean ± SEM. *N* = 4. The vasculature was outlined by intravascular labeling with lectin (*green*)

We then explored whether apoE in CSF enters brain via the perivascular space. First, we intracisternally administered Alexa-647 labeled human-lipidated apoE4 (apoE4-647) and a reference molecule, cascade blue-labeled dextran (CB; 10 kDa, fixable) (Fig. 2a), to establish the appropriate time point for further studies. At various post-injection times, mice were perfusion-fixed with 4% paraformaldehyde (PFA) followed by horizontal (rather than coronal) brain section. These procedures allowed us to evaluate apoE distribution as a function of distance from the vessel wall. To clearly visualize the entire vasculature, lectin, a vascular marker, was administered intravascularly. Radial distribution pattern of both molecules (CB > apoE4-647) was seen around arterial vessels but not veins (Additional file 2: Figure S2A-B). The radial distribution increased progressively with post-injected time with CB distributing, due to the bulk flow of ISF, further from the vessel wall than that of apoE4-647 (Fig. 2b-e). Surprisingly, the distribution of apoE4-647 was limited and quickly approached equilibration. Interestingly, the distribution of non-lipidated apoE4-647 was greater than that of lipidated apoE4-647 (Additional file 3: Figure S3A-H), which may explain the higher ISF clearance of non-lipidated apoE isoforms compared to their lipidated counterparts [6]. The distance of apoE distribution from the vessels wall was then standardized to that of the reference molecule (Fig. 2f). We selected 15 min after the intracisternal injection to study the CSF inflow into brain since it appears that equilibration occurs at about 25 min and to allow for changes, either increase or decrease.

**Fig. 3:**
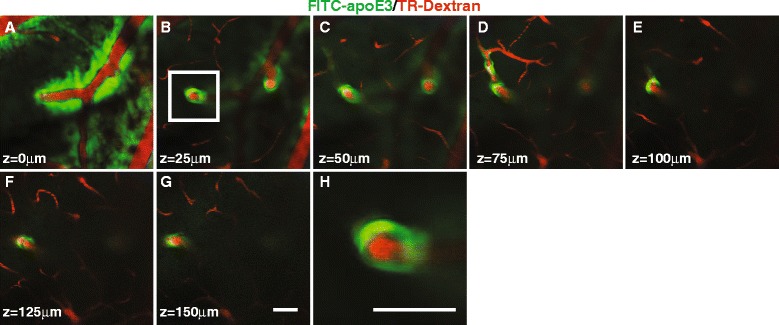
ApoE periarterial inflow using in vivo 2-photon imaging (**a**) Pial surface arterial vessels showing apoE3-FITC within the perivascular space of arteries. **b**-**g** ApoE3-FITC along the perivascular space of penetrating arterial vessel at various depths below the brain surface. **h** Magnified image of the white boxed area in (panel **b**) (scale bar 50 μm). Following intracisternal injection (5 μL at 1 μL/min) of apoE3-FITC images were acquired every 25 μm below the brain surface at 15 min. The scale bar in (panel **g**) is 50 μm. Panels **a**-**g** are at the same magnification. The vasculature was identified by intravenously injected Texas Red-dextran (70 kDa)

**Fig. 4:**
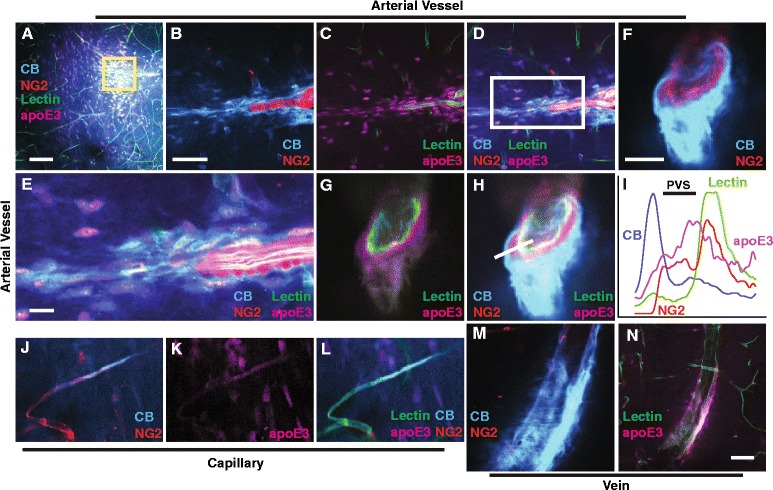
ApoE is cleared from brain along perivascular pathways **a** Low magnification image showing the distribution of intracisternally injected apoE3-647 and cascade blue (10 kDa) in brain 15 min after their injection (5 μL at 1 μL/min). **b**-**d** Magnified images of the boxed area in (panel **a**). **e** Magnified images of the boxed area in (panel **d**). **f**-**h** Cross section of a cortical penetrating artery showing the perivascular flow of apoE3-647 and cascade blue. **i** Intensity graph of the various fluorescent markers across the white line drawn in (panel **h**). **j**-**l** Presence of apoE3-647 and cascade blue in a representative cortical capillary 60 min after intraparenchymal injection (caudate nucleus, 0.5 μL at 0.1 μL/min). **m**-**n** Presence of apoE3-647 and cascade blue in a representative deep cerebral parenchymal vein 60 min after intraparenchymal injection (caudate nucleus). Intracisternal apoE3-647 (*magenta*) and cascade blue (*blue*) entered brain via the penetrating cortical arterial vessels (*red*) and distributed within the parenchyma. Panels **a**-**h** are images after intracisternal injection of apoE3-647 and cascade blue in NG2-DsRed reporter mice. Lectin-FITC was injected intravascularly to identify vessels. Panels **j**-**n** are images after intraparenchymal (caudate nucleus) injection of apoE3-647 and cascade blue in NG2-DsRed reporter mice. Scale bars: A = 100 μm, B-D = 50 μm, E-H and J-N =10 μm. PVS = perivascular space

To confirm the highway by which CSF-derived apoE enters into brain, we used two-photon laser scanning microscope to visualize the pathway along vessels through a closed cranial window in anesthetized mice. The cerebrovasculature was clearly visualized by intravascular injection of the BBB impermeable Texas red-dextran (70 kDa). Intracisternal injected apoE3-647 entered the perivasculature space along surface arteries and penetrating arterioles (Fig. 3a-h), as reported for other molecules [26, 27]. Inflow of CSF-derived apoE3-647 at 15 min after the intracisternal injection was seen within the periarterial space at various depths below the brain surface (Fig. 3b-h). We then used ex vivo brain sections to confirm these results and apoE’s presence in other parts of the vasculature, such as capillaries and veins. ApoE3-647 and CB (10 kDa) were intracisternally injected and lectin was administered intravascularly to label vessels in NG2-DsRed reporter mice. We confirm the presence of apoE3-647 and CB in the perivascular space of arterial vessels and their distribution into the parenchyma (Fig. 4a-h). To confirm the clearance routes, apoE3-647 and CD was injected (0.5 μL at 0.1 μL/min) into the parenchyma (caudate putamen) and after 60 min the brain sections analyzed for the location of these molecules along vessels. ApoE3-647 and CB was present along capillaries (Fig. 4i-k) and deep cerebral parenchymal veins (Fig. 4l-m). Clearance of apoE was confirmed by using ^125^I-apoE3 (10 nM) and ^125^I-apoE4 (10 nM), as shown (Additional file 3: Figure S3I). Iodination (^125^I-apoE) does not affect clearance when compared to unlabeled apoE [44]. Furthermore, FITC labeling of apoE3 does not affect it clearance when compared to ^125^I-apoE3 (Additional file 3: Figure S3J).

**Fig. 5:**
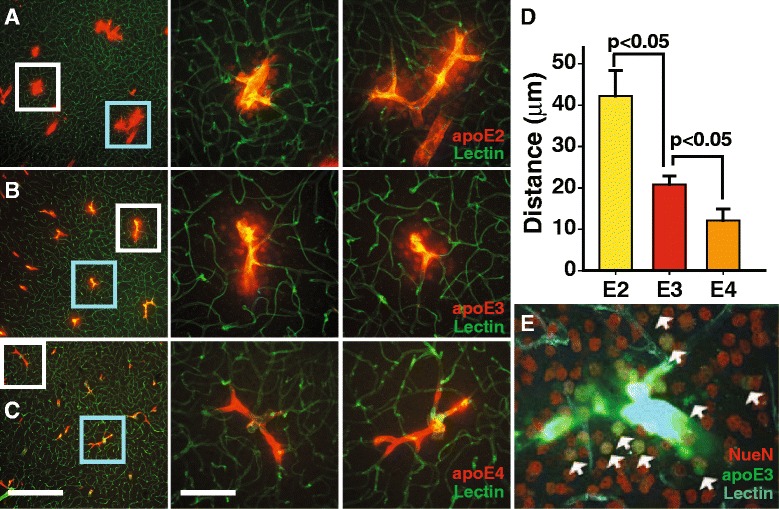
ApoE-isoform specific distribution around arteries. **a-c** Representative images of apoE2, apoE3 and apoE4 (colored *red*) radial distribution 15 min following their intracisternal injections. Scale bar 100 μm for the left panels and 50 μm for the middle/right panels. **d** Quantification of the distribution distance from the arteries. Values are mean ± SEM. *N* = 5–9. **e** FITC-apoE3 in the CSF binding to NeuN- positive neurons (*red*) in the vicinity of arteries. *White arrows* point to apoE3/NeuN- double positive cells (the image is 100 μm wide). The vasculature was outlined by intravascular labeling with lectin (*blue*)

Next, the distribution of the three apoE isoforms was compared to establish whether there are isoform-specific effects. Unexpectedly, the radial distribution from the arterial wall was apoE isoform specific, with apoE2 and apoE3 having greater radial flow than that of apoE4 even though the particle size of each isoform was similar and the same amount of apoE was injected (Fig. 5a-d). The distribution radius of apoE2 was 1.75-fold greater than apoE3, which in turn was 2-fold greater than apoE4. These observations are consistent with the hypothesis that apoE binds avidly to apoE receptors and that the brain retention of apoE4 is greater that apoE3 and apoE2 [6, 53–55], and thus, bulk flow of apoE4 is much more restricted compared to apoE2. The distribution distance of CB was not affected by the apoE isoforms (Additional file 4: Figure S4). To determine whether apoE4 degradation contributed to its restricted distribution, radiolabeled apoE (10 nM) was intracisternally injected and after 30 min the brain was removed and TCA-precipitable radioactivity assessed. The level of TCA-precipitable ^125^I-apoE (intact apoE) in the brain was similar for each isoform (Additional file 5: Figure S5). Thus, there was no significant difference in apoE degradation between the isoforms, as reported for up to 300 min [6, 44]. To establish whether CSF apoE is an important source of neuronal apoE, we intracisternally injected FITC-apoE3, and after 15 min, perfused and PFA fixed the brain. Brain sections were then immunolabeled with NeuN, a neuronal specific nuclear biomarker that is used extensively. The CSF injected FITC-apoE3 was associated with peri-arterial neurons (NeuN-positive cells; Fig. 5e). Interestingly, apoE was associated with the nucleus, as reported [56, 57]. Intracisternal injected FITC-ovalbumin (45 kDa) or FITC-dextran (40 kDa, fixable) was not present in the nucleus of neurons (Additional file 6: Figure S6 A-H). However, intracisternal injected unlabeled apoE3, detected with a specific anti-human apoE antibody, was present in the nucleus (Additional file 6: Figure S6 I-L). Thus, CSF apoE enters the brain parenchyma via the periarterial space, taken up by neuronal cells and some of the apoE enters the nucleus.

**Fig. 6:**
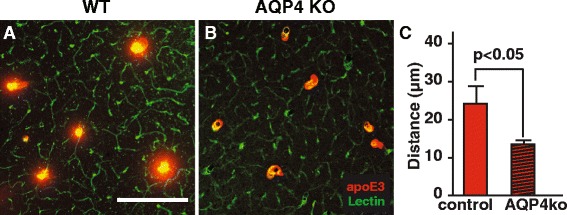
CSF apoE distribution in brain is reduced in *Aqp4* ^−/−^ mice. Representative images of FITC-apoE3 radial distribution from the arteries in littermate controls (**a**) and *Aqp4* ^−/−^ (**b**) mice. **c** Quantification of the distribution distance from the arteries. Values are mean ± SEM, *N* = 5 mice per group

Since AQP4 mediates ISF distribution we used *Aqp4* ^−/−^ mice, as reported in similar studies [26]. The radial distribution of apoE3 was considerably reduced in the *Aqp4* ^−/−^ mice compared to littermate controls (Fig. 6a-c). Similarly, cascade blue labeled dextran (10 kDa) distribution was reduced (Additional file 7: Figure S7A-B). In addition, in *Aqp4* ^*−/−*^ mice, the brain uptake of ^125^I-apoE2, ^125^I-apoE3 and ^125^I-apoE4 (10 nM), after their intracisternal injection, was equally suppressed (Additional file 7: Figure S7C). Similarly, ^14^C-inulin brain uptake was reduced and not affected by apoE isoforms (Additional file 7: Figure S7D). A previously report has shown that other CSF tracers, such as Aβ and ovalbumin, were reduced by 60% in *Aqp4* ^−/−^ mice [26]. Thus, CSF apoE is distributed into brain parenchyma by glymphatic fluid transport in a radial pattern around arterial vessels.

**Fig. 7:**
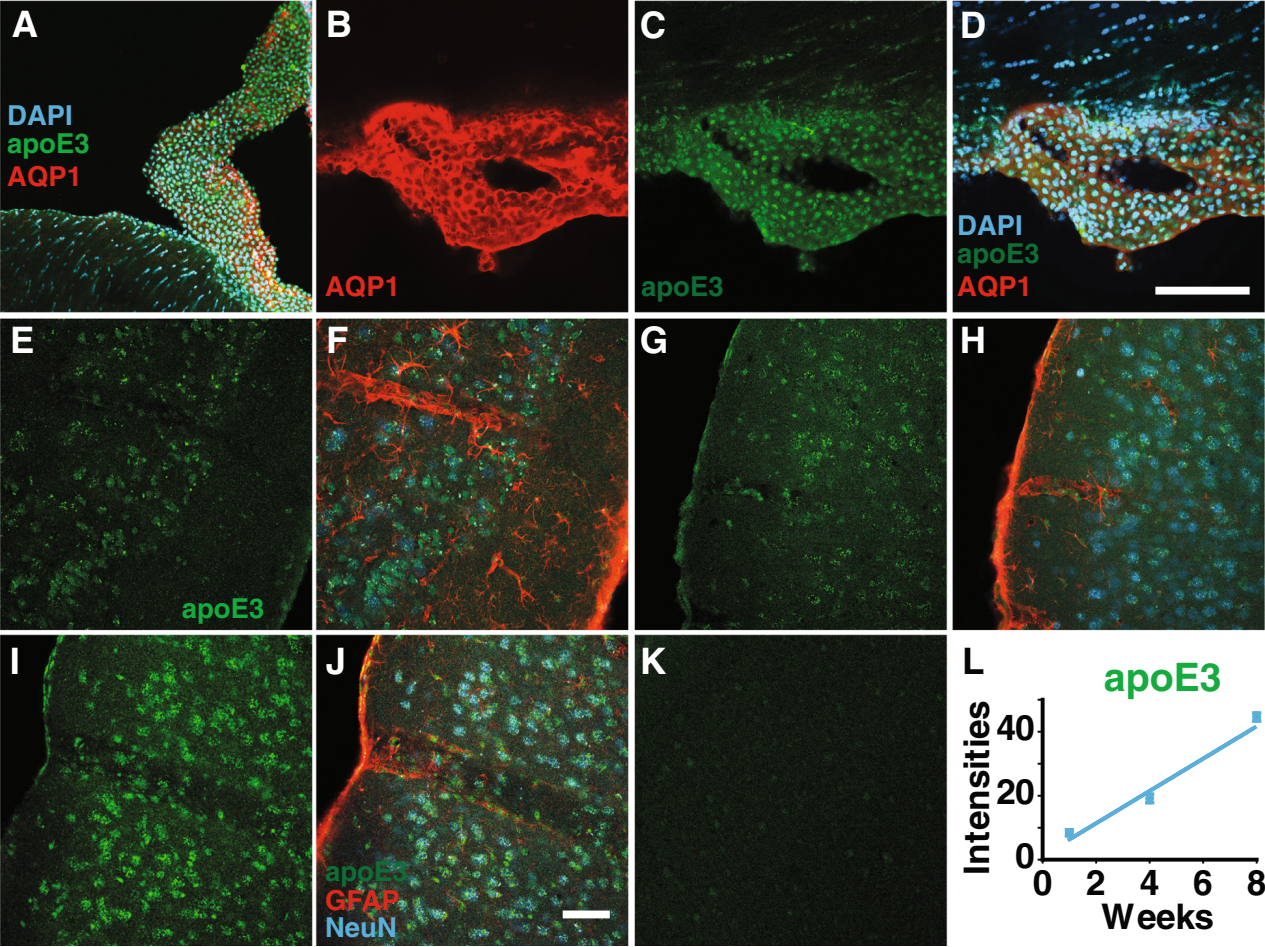
ApoE3 levels in brain cells increase following lenti-apoE3 transduction of the choroid plexus. **a** Lenti-apoE3 effectively transduced the choroid plexus and ependymal layer at 1 week post-transduction. ApoE3 immunolabeled with a human specific anti-apoE antibody. **b**-**d** Choroid plexus expression of apoE3 at 8 weeks post-transduction. Scale bar 100 μm. AQP1 (*red*; **b**); apoE3 (*green*; **c**) and merged images (**d**). **e**-**j** Parenchymal human apoE3 (*green*) at 1 (**e**-**f**), 4(**g**-**h**) and 8 (**i**-**j**) weeks post-transduction. Scale bar 100 μm. Neurons: NeuN-positive cells (*blue*). Astrocytes: GFAP -positive cells (*red*). **k** Human specific anti-apoE does not react with endogenous mouse apoE. **l** Quantification of apoE3 intensities in neurons (apoE co-localization with NeuN-positive cells) with post-transduction time. Lenti-apoE3 delivered intraventricularly (3 μL lenti- apoE (4.06 × 10^8^ TU/ml) and after 1 to 8 weeks the brains were perfusion fixed (PFA) followed by immunolabeling of brain sections. Representative images from 5 mice per group. Values are mean ± SEM. *N* = 5

ApoE, produced by the choroid plexus and secreted into the CSF, could be an important source of ISF apoE, since CSF is circulated through the brain parenchyma by glymphatic fluid transport [26, 27, 29, 30]. To test whether apoE produce by the choroid plexus is delivered to the brain parenchyma, we transduced the choroid plexus with a lentivirus encoding a human apoE construct (lenti-apoE3) and mapped apoE3 distribution with a human specific apoE antibody. First, we injected lenti-EGFP into the lateral ventricle and found that it intensely transduced the choroid plexus and ependymal cells but not parenchymal cells (Additional file 8: Figure S8A-E). We confirmed that endogenous apoE is highly expressed in the choroid plexus (Additional file 9: Figure S9). We next found that lenti-apoE3 delivered intraventricularly intensely transduced the choroid plexus and ependymal layer, as seen for lenti-EGFP (Fig. 7a-d; Additional file 8: Figure S8A-E). In addition, apoE3 neuronal uptake increased with post-transduction time (Fig. 7e-l). To confirm that the lentivirus did not transduce neurons and astrocytes, we injected a mixture of lenti-EGFP and lenti-apoE3 into the lateral ventricle and after 4 weeks analyzed brain sections. In these experiments a mixture of both lenti-EGFP and lenti-apoE3 was used since the IRES bicistronic expression of EGFP is low compared to that of apoE3 in this expression cassette. Thus, to get a good EGFP signal we used a mixture of both lenti-EGFP (to identify the transduced cells only) and lenti-apoE3 (to identify the expressed apoE3 by immunolabeling) to achieve a more rigorous test of the transduced cells. In Additional file 10: Figure S10, EGFP expression (lenti-EGFP) shows the transduced cells, i.e., only the choroid plexus and ependymal layer. In contrast, human apoE3 secreted by the transduced cells (lenti-apoE3) was detected by immunolabeling, and shown to be present in these regions and in the brain parenchyma (Additional file 10: Figure S10 A-H). Thus, the lentivirus was taken up only by local cells, the choroid plexus and ependymal cells, as shown for viral transduction [49, 58].

**Fig. 8:**
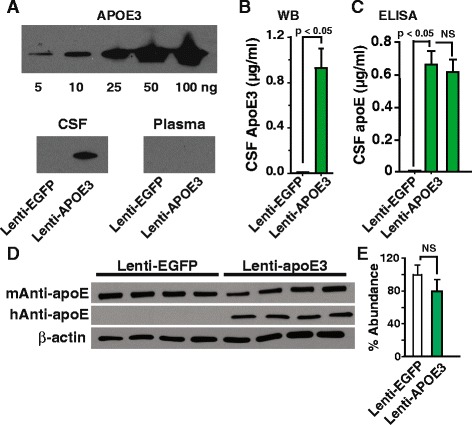
Increased CSF and brain apoE3 levels in lenti-apoE3 transduced choroid plexus. **a** Representative Western blots of apoE3 standards (*top*) and apoE3 in CSF and plasma from lenti-EGFP (controls) and lenti-apoE3 transduced mice. Quantification of CSF apoE3 levels by Western blot analysis at 4 weeks post-transduction (**b**), and by ELISA at 4 (*left*) and 8 (*right*) weeks post-transduction (**c**). **d** Levels of brain endogenous apoE and human apoE3 in the transduced mice. **e** Quantification of mouse apoE levels by Western blot analysis. Values are mean ± SEM. *N* = 4-5. Intraventricular (unilateral) injection of 3 μL lenti-human apoE (4.06 × 10^8^ TU/ml) or 3 μL lenti-EGFP (10^12^ TU/ml). At 4 and 8 weeks post-transduction samples of CSF, plasma and brain were collected

In addition, human apoE3, determined by Western blot analysis and by ELISA with a human specific anti-apoE antibody, was present in CSF collected from the lenti-apoE3 transduced mice (Fig. 8a-c). Furthermore, primary culture of mouse choroid plexus epithelial cells secreted similar levels of apoE compared to primary culture of mouse astrocytes (Additional file 11: Figure S11A-B). The purity of the cultured choroid plexus epithelial cells and astrocytes were typically >99% (Additional file 11: Figure S11C-F). Mouse brain apoE levels were unchanged in the lenti-apoE3 transduced mice compared to that of the lenti-EGFP transduced mice (Fig. 8d-e). While an accurate determination of human apoE levels cannot be compared to mouse apoE since different antibodies were used, the estimated brain apoE3 levels in the transduced mice represent about 25% of the endogenous mouse apoE (Fig. 8d-e). Taken together these data show that apoE3, secreted by the choroid plexus into CSF, was delivered to the brain via glymphatic fluid transport [26].

**Fig. 9:**
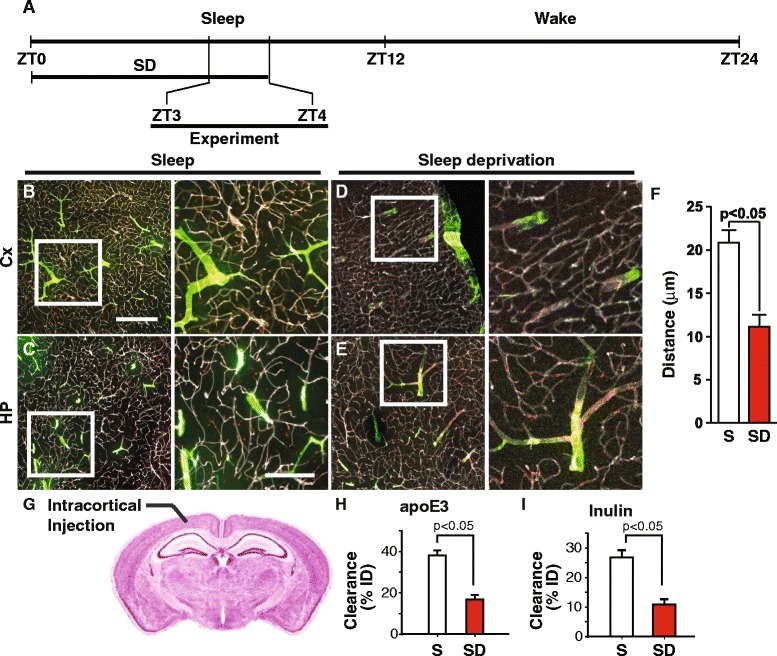
Sleep deprivation reduces the apoE3 arterial radial diffusion and clearance. **a** Schematic representation of the experimental design. Representative images at low (*left*) and high (*right*) magnification of the cortex (**b**) and hippocampus (**c**) in mice on normal sleep cycle. Representative images at low (*left*) and high (*right*) magnification of the cortex (**d**) and hippocampus (**e**) in sleep deprived mice. **f** of the distribution distance from the arteries in the cortex of control (sleep (S); clear column) and sleep deprived (SD; red column) mice. Fluorescently-tagged apoE3 was injected into cisterna magna of NG2dsRed reporter mice and perfusion fixed (PFA) at 15 min. The vasculature was outline by lectin (gray). ApoE, green; NG2-dsRed, red. Scale bars 100 μm (A-D, *left*), 50 μm (A-D, *right*). **g** Schematic diagram showing the intracortical injection site. **h**-**i** ^125^I-ApoE3 and ^14^C-inulin clearance from the frontal cortex in the sleep and sleep deprived states. Values are mean ± SEM. *N* = 5 mice per group

We have shown that glymphatic inflow of CSF into brain was reduced in mice that were awake compared to the sleep state [27]. Thus, we reasoned that lack of sleep would reduce CSF inflow into brain and thereby, decrease the delivery of CSF-derived apoE to brain. Mice were sleep deprived for 3 h and the flow of CSF-derived apoE3 into brain determined at zeigeber time 3 (ZT3; Fig. 9a). The analysis showed that sleep deprivation significantly reduced the radial distribution of apoE3 around arterial vessels compared to mice on normal sleep cycle (Fig. 9b-f). Similarly, the radial distribution of cascade blue labeled-dextran was reduced in sleep deprivation (Additional file 1: Figure S12A-B). In addition, inflow of CSF-derived ^125^I-apoE2, ^125^I-apoE3 and ^125^I-apoE4 (10 nM) and ^14^C-inulin into brain was reduced to the same degree during sleep deprivation (Additional file 12: Figure S12C-D). Interestingly, clearance of ^125^I-apoE3 (10 nM) and ^14^C-inulin from the ISF was also reduced in the sleep deprived mice compared to those in the sleep state (Fig. 9g-i).

## Discussion

The analysis presented here shows, for the first time, that choroid plexus/CSF-derived apoE is delivered into brain, via glymphatic fluid transport, in an apoE-isoform specific (apoE2 > apoE3 > apoE4) radial distribution pattern around penetrating arterial vessels, but not veins. *Aqp4* ^*−/−*^ mice exhibited a significant decrease in the apoE distribution in brain due to the reduced ISF bulk flow and apoE-isoform distribution cannot be resolved, i.e., loss of the isoform specific distribution. Perhaps most interestingly, sleep deprivation was associated with a striking reduction in CSF delivered apoE to the parenchyma, and a loss of apoE-isoform specific distribution into brain also due to the reduced ISF bulk flow. These observations of radial apoE distribution from arterial vessels of CSF-derived apoE support the novel concept of a macroscopic distribution mechanism of compounds secreted by the choroid plexus into brain. Speculating, failure of glymphatic CSF inflow into brain, as in sleep deprivation, may, in the long-term, contribute to apoE related disorders and eventually neurodegenerative diseases [59–62].

It’s well established that the choroid plexus is a major source of CSF [63–65]. However, the choroid plexus also secretes essential substances into CSF, such as transthyretin, which binds thyroxine to facilitate its distribution within CSF and brain [66, 67]. In addition, blood-derived molecules, such as apoA1, enter brain via the choroid plexus [68]. Glymphatic fluid transport may be the medium for the delivery of these molecules from CSF to brain. There are a number of findings that collectively strongly indicates that the choroid plexus also regulates the levels of apoE. In *Abca1* ^−/−^ mice, brain apoE levels were reduced [11, 69], but CSF levels were almost completely suppressed [11]. Liver X receptors (LXR) are sensors of cellular cholesterol that regulate the expression of apoE, (ATP-binding cassette transporter A1 (ABCA1) and other genes involve in lipid metabolism. Activating LXR in the CNS increases CSF levels of apoE and cholesterol more profoundly compared to brain levels. LXR activation increases apoE expression in the choroid plexus [21], and ABCA1 levels and cholesterol release from the choroid plexus [20]. Despite these observations the significance of this source of apoE to brain parenchyma apoE levels has been ignored. Since the glymphatic system allows CSF to enter brain by bulk flow, apoE in the CSF may contribute to parenchymal apoE. While neurons can synthesize cholesterol they may need additional cholesterol delivered in the form of apoE-associated cholesterol [9]. In the Niemann-Pick type C1 mouse, which has a mutant NPC1 gene, cholesterol accumulates in the late endosome and lysosomes in neurons, suggesting uptake of apoE-associated cholesterol via clathrin-coated pit pathway [70, 71]. Interestingly, the expression of apoE in astrocytes, the main producer of brain apoE, is not uniform in brain [17].

ApoE, a cargo transporter and a cell signal molecule, has essential and diverse role in the CNS [7]. It’s involved in neuritic growth, synaptic plasticity, regeneration and remyelination of axon and cognition [72]. As a cargo transporter it distributes cholesterol within body fluids and essential for the delivery of cholesterol and lipids to cells [25]. Our data demonstrated that CSF may serve as a medium for the delivery of apoE to brain via glymphatic fluid transport, as reported for other solutes, such as Aβ [26, 30], lipophilic molecules [29] and tau [73]. Within brain, ISF is distribute by bulk flow, which has been established using inert molecules that is not significantly taken up by receptor mediated transport into cells, degraded or transported across the BBB [74–86]. We have confirmed bulk flow of ISF since inert molecules of different molecule weights (mannitol 180 Da vs dextran 10,000 Da and inulin 6,000 Da vs dextran 40,000 Da) are cleared at the same rate, i.e., bulk flow [26, 46]. Earlier reports suggested that molecules transported along the peri-vascular space occurs in the opposite direction to cerebral blood flow, i.e., brain to CSF [50, 51, 87, 88]. Flow of ISF from brain to CSF may occurs along the vascular basement [86]. While it is possible for bidirectional trafficking along the vessel [86], more investigation will be needed to clarify this fully.

What is the importance of CSF-derived apoE to brain apoE since astrocytes produce apoE? Primary astrocytes in culture secrete apoE [14], and this could be taken up into local neurons by receptor-mediated endocytosis [89]. Our data show that CSF-derived apoE is taken up by neurons, in vivo. Thus, astrocyte-derived apoE is not completely blocking CSF-derived apoE from accessing these cells. In other words, astrocytic apoE is not saturating all apoE receptors on the cell surface that would prevent the uptake of CSF-derived apoE. Thus, CSF-derived apoE may be an important source of brain apoE. The glymphatic fluid transport of CSF-derived apoE may also prevent dilution and wash out of astrocyte-secreted apoE, and facilitate a wider distribution of apoE within the parenchyma. In addition, glymphatic re-circulation of apoE may contribute to its slow turnover rate [90], and retention within brain [6].

ApoE polymorphism influences its structure and function [4]. Compared to apoE2 and apoE3, the arginine residue at position 112 of apoE4 may promote interactions between the N-terminal (receptor-binding region) and C-terminal (lipid-binding region) domains, which causes instability [4, 24]. Thus, the apoE isoform specific radial distribution of CSF-derived apoE around arteries could reflect its cellular uptake, which restricts the flow of apoE within the ISF, especially apoE4. Cells along the glymphatic pathways express many types of apoE receptors, such as low-density lipoprotein receptor (LDLR) and LDLR-related protein 1 (LRP1). These receptors are expressed in many cell types, including vascular smooth muscles, neurons, glia and endothelial cell [91–93]. Lipidated apoE, the normal form of apoE and the apoE used in the present studies, avidly binds to apoE receptors [6, 53, 94, 95]. There are subtle differences in the binding constants of each apoE isoform to apoE receptors [94]. For example, apoE2 binds LDLR 50- to 100-times weaker than apoE3 and apoE4 and this may explains it greater distribution radius [96]. Interestingly, apoE isoform specific retention within brain (apoE4 > apoE3 > apoE2) [6] corroborate the data obtained for their radial distribution from the arterial wall (apoE4 < apoE3 < apoE2).

Glymphatic activity is regulated by the sleep/wake state: with inflow of CSF into brain greater during the sleep state compared to wakefulness [27]. Sleep is also required for greater bulk flow of brain ISF and clearance of solutes [27], consolidation of memory and plasticity [60]. Many studies have focused on the effect of sleep deprivation on synaptic plasticity at the molecular and electrophysiological levels, and on the structural aspects of brain regions associated with learning and memory, such as the hippocampus [97]. To our knowledge there are no studies on the effect of sleep deprivation on brain-wide delivery of CSF-derived apoE. While many factors may contribute to the mechanism for the observed changes during sleep eprivation [98], increased noradrenaline levels [99, 100] may play a role in reducing glymphatic fluid transport, as reported [27]. The causes of sleep deprivation are complex and multifactorial, which involves many biological factors, including metabolic and neurotransmitter changes [101, 102]. We use a milder procedure to induce sleep deprivation to minimize major changes. CSF inflow into brain via the periarterial space and ISF clearance are reduced during wakefulness compared to the sleep state, and antagonist of norepinephrine induced a sleep-like response in CSF inflow [27]. It is possible that norepinephrine released during ‘sleep deprivation stress,’ even though this was minimized, may contribute to the reduced CSF inflow and ISF clearance. Interstitial levels of norepinephrine are increased in the unstrained mice compared to mice in the sleep-like state [27]. Sleep deprivation may increase the sympathetic tone [103], especially in mice that are sleep deprived for the first time and when they want to sleep (light switched off). Norepinephrine may cause vasoconstriction of pial arteries [104], and this may contribute to the reduced CSF inflow during sleep deprivation. Recently, it was show that Aβ40 in CSF caused a reduction in CSF inflow via the periarterial space, possible due to vasoconstriction of pial arteries [30]. Thus, impaired CSF inflow during sleep deprivation may be due to vasoconstriction of the pial vessels, and this may also contribute to the reduced glymphatic fluid transport. Speculating, failure in the delivery of CSF-derived apoE to brain during sleep deprivation may limit the brain-wide distribution of apoE and other essential substances to brain cells, which may, in the long-term, contribute to apoE related dysfunction [105–108]. Further work is needed to explore other possible mechanisms.

The implications of these findings are that reduced glymphatic inflow into brain, as seen in sleep deprivation, the wake state [27], aging [42] and in brain injury and trauma [46, 73], could reduce the delivery and distribution of apoE to brain, which may lead to apoE related disorder in the long-term. However, a dilemma is that the apoE isoform specific restriction to arteries may lead to the development of cerebral amyloid angiopathy (CAA), vascular dementia and AD. The apoE4 isoform predisposes heightened susceptibility to AD and CAA [109, 110]. Intriguingly, the reduced glymphatic perivascular flow with aging and brain injury may facilitate the development of CAA due to the slower transit time that will cause greater cellular binding/update of apoE, especially apoE4. While apoE2 has the longest distribution it was reported to be associated with greater incidence of micro hemorrhage as was apoE4 [111–113].

**Fig. 10:**
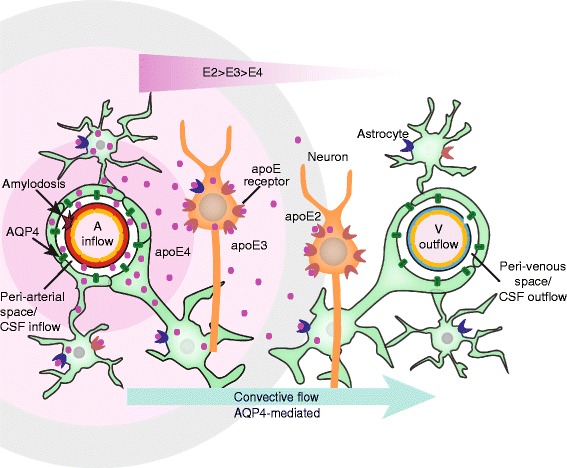
Schematic diagram of our working model for apoE delivery and distribution by the glymphatic system. This schematic diagram shows the parenchymal distribution of CSF-derived apoE isoform-specific convective flow/bulk flow pattern (apoE2 > apoE3 > apoE4) around an artery. CSF-derived apoE is present on the vessel wall and around the artery. The apoE distribution is shown as a circle around the artery for simplicity but its bulk flow (unequal distribution around the artery). Within the brain there are many cells, such as neurons, astrocytes that express many membrane bound receptors that avidly binds apoE. Symbols: A - penetrating arteriole; V - vein;  - apoE;  **-** AQP4**;** There are many different types of apoE receptors, such as  **-** apoE receptor mainly on astrocyte and  - apoE receptor mainly on neuron

Gene therapy targeting the choroid plexus to enhance the production of apoE2 or to produce apoE2 in apoE4 allele carriers may extend the apoE distribution radius [114]. Viral transduction was effective in selectively transducing the choroid plexus and ependymal cells [49, 58]. The use of lentivirus to target the choroid plexus, via intraventricular injection, could minimize tissue injury due to intra-cerebral injections [115]. Further work is needed in a model of AD to evaluate the effect of this intervention on glymphatic fluid transport and in preventing cognitive decline. Despite decades of research it is still unclear whether therapies to lower or increase brain cholesterol is require to treat AD [49, 116, 117]. Altering CSF apoE levels may provide better insights into the level and form of apoE require for its sustained beneficial effects. Fig. 10 shows a schematic diagram of our working model.

## Conclusion

The analysis shows, for the first time to our knowledge, the following:

1. Choroid plexus/CSF-derived apoE is rapidly delivered into brain, via glymphatic fluid transport, in an apoE isoform specific (apoE2 > apoE3 > apoE4) radial distribution pattern around arterial vessels, but not veins.
2. *Aqp4* ^*−/−*^ mice exhibited a significant decrease in the apoE distribution in brain.
3. ApoE3, delivered to the choroid plexus by lentivirus, was present in the CSF and delivered to brain cells via the perivascular space.
4. Sleep deprivation was associated with a reduction in CSF inflow into brain and clearance from the parenchyma.
5. These observations of radial apoE distribution from arterial vessels of CSF-derived apoE support the novel concept of a macroscopic distribution mechanism of compounds secreted by the choroid plexus into brain via the CSF inflow along the periarterial space.
6. Speculating, failure of this glymphatic CSF inflow into brain, as in sleep deprivation, may, in the long-term, contributes to apoE related dysfunction.

## Additional files

Additional file 1: Figure S1. More GFAP-positive astrocytes on the arterial wall than that on veins. a-e) Representative images of GFAP-positive astrocytes on the wall and around arteries and veins. The vasculature was outline by lectin (green) in NG2dsRed reporter mice and perfusion fixed (PFA) at 15 min. A) Astrocytes (GFAP, blue), B) Vessels (lectin-FITC, green), C) arteries (NG2-dsRed, red), D) endogenous apoE (purple) and E) Merged images. Images of GFAP-positive astrocytes around an artery (identified by the expression of NG2-DsRed and vasculature staining with lectin) as shown in the yellow box in C (F) and a vein (identified by the lack of NG2-DsRed expression but with the vasculature stained with lectin) as shown in the white box in C (G). Scale bars: 100 μm (A-E), 50 μm (F-G). Arrow heads (panel F) identify some astrocytic endogenous apoE. (EPS 7646 kb)
Additional file 2: Figure S2. ApoE4 radial distribution around arteries is shorter than that of dextran. A) Representative images of radial distribution around arteries but not veins for dextran-cascade blue (inert reference molecule, CB) and apoE4-Alexa647 (apoE-647, purple) after 10 min post-injection in NG2Ds-Red (NG2, red) reporter mice. The vasculature was outlined by intravascular labeling with lectin (green). Scale bar = 100 μm. Arteries (yellow arrow head). Veins (blue arrows). B) A custom made image J plugin was used to generate the mean radial distribution of cascade blue labeled dextran (Dextran-CB, blue) and apoE4-Alexa647 (colored red) from the lectin stained vessel wall (green). Distribution from the vessel wall was determined at 1000 intensity units and maximum intensity indicate saturation at 4096 intensity units (2^12^ – 12 bit image depth). (EPS 32092 kb)
Additional file 3: Figure S3. Radial distribution around arteries is greater for non-lipidated than lapidated apoE4. A-H) Representative images (from 3 to 4 mice) of radial diffusion of non-lipidated (A-D) and lipidated apoE4-Alexa647 that was colored green (E-H) at 10 min after their intracisternal injection in NG2ds-Red (NG2, red) reporter mice. The vasculature was outlined by intravascular labeling with lectin (colored blue). Scale bar = 100 μm. I) Clearance of ^125^I-apoE3 and ^125^I-apoE4 (10 nM) 90 min after their intracortical injection (0.5 μL over 5 min). J) FITC-apoE3 (10 nM) or ^125^I-apoE3 (10 nM) ISF clearance were similar 90 min after their intracortical injection. Values are mean ± SEM, *n* = 5-6 mice. (EPS 26205 kb)
Additional file 4: Figure S4. ApoE isoforms does not affect cascade blue-dextran radial distribution. Similar radial distribution distances of dextran-cascade blue (CB) in the presence of apoE2 (E2), apoE3 (E3) and apoE4 (E4) at 15 min after their intracisternal injection. Values are mean ± SEM, *N* = 5-9. (EPS 564 kb)
Additional file 5: Figure S5. No differences in apoE degradation in brain. Brain levels of TCA-precipitable ^125^I-apoE isoforms (intact) 30 min after their intracisternal injection. Values are mean ± SEM, *N* = 5. (EPS 567 kb)
Additional file 6: Figure S6. ApoE in the nucleus of neurons but not FITC-dextran or FITC-ovalbumin. A-C) FITC-ovalbumin (45 kDa, green) is not present in the nuclei of NeuN-positive neurons (red). D) Magnified image (boxed area in panel C) showing the NeuN-positive nucleus lacking FITC-ovalbumin labeling. E-G) FITC-dextran (40 kDa, green) is not present in the nuclei of NeuN-positive neurons (red). H) Magnified image (boxed area in panel G) showing the NeuN-positive nucleus lacking FITC-dextran labeling. Scale bar: 100 μm (A-C and E-G), 50 μm (D and H). I-K) Unlabeled apoE3 (10 nM) injected intracisternally was taken up by NeuN-positive nucleus at 30 min. L) Magnified image of the boxed area in panel K. Scale bars 25 = μm. (EPS 48178 kb)
Additional file 7: Figure S7. Reduced cascade blue radial distribution and apoE inflow into brain in *Aqp4* ^−/−^ mice. Representative images of cascade blue dextran (10 kDa, blue) radial distribution in littermate controls (A) and *Aqp4* ^−/−^ (B) mice. Cascade blue dextran was injected intracisternally, and the mice perfusion-fixed at 15 min. The vasculature was outline by lectin (green). Scale bar is 100 μm. C) Inflow of CSF-derived ^125^I-apoE2 (yellow column,10 nM), ^125^I-apoE3 (red column, 10 nM) and ^125^I-apoE4 (orange column, 10 nM) into brain was suppressed in *Aqp4* ^*−/−*^ mice. D) Inflow of CSF-derived ^14^C-inulin into brain was suppressed in *Aqp4* ^*−/−*^ mice. Values are mean ± SEM. *N* = 6 mice per group. (EPS 14922 kb)
Additional file 8: Figure S8. Lenti-EGFP effectively transduced the choroid plexus. A) Lenti-EGFP unilateral injected into the lateral ventricle preferentially transduced the choroid plexus and ependymal layer but not brain parenchyma at 1 week. DAPI (blue); EGFP (green). B-E) Lenti-EGFP expression in the choroid plexus at 1 week. Images are DAPI (blue; B), AQP1 (red; C); EGFP (green; D) and merged images (E). Scale bar 50 μm. Intraventricular injection of 3 μL lenti-EGFP (10^12^ TU/ml). *N* = 5. (EPS 75360 kb)
Additional file 9: Figure S9. Endogenous apoE expression in the choroid plexus. Immunolabeling of mouse endogenous apoE in the choroid plexus. Note the high apoE expression level in choroid plexus. Blue, DAPI; green, apoE; red, NG2-dsRed; gray, Lectin. (EPS 824 kb)
Additional file 10: Figure S10. Lenti-apoE3 transduced the choroid plexus but not the parenchyma cells. A-H) Representative images showing transduction in the choroidal cells of lenti-apoE3 and lenti-EGFP at 4 weeks after intraventricular delivery. A) Merged image showing choroid plexus (CP; scale bar, 100 μm). B) EGFP (green); C) apoE3 (magenta); D) AQP1 (red); E) merged images of B, C and D (scale bar 50 μm). Arrows show apoE3 in choroidal cells in C and E. F-H) cortex (Cx) showing no transduction (no EGFP) (F, scale bar 200 μm) but the presence of apoE3 (G-H). H, scale bar 50 μm. Lenti-apoE3/lenti-EGFP mixture was delivered intraventicularly (3 μL lenti-human apoE (4.06 × 10^8^ TU/ml) and lenti-EGFP (10^12^ TU/ml) and after 4 weeks the brains were perfusion fixed followed by immunolabeling. (EPS 41288 kb)
Additional file 11: Figure S11. Cultured primary mouse choroid plexus epithelial cells secrete apoE. A) Representative apoE images from Western blots of the conditional medium from choroidal epithelial cells and astrocytes. B) Primary cultured choroidal epithelial cells (CP) produced similar levels of apoE as astrocytes (astro). Values are mean ± SEM, *N* = 4. C) Representative image of AQP1, a marker of choroid plexus epithelial cell, expression in the cultured primary mouse choroid plexus. Lack of significant immunolabeling of NeuN (neurons; D) and GFAP (astrocytes; E) in the cultured CP epithelial cells. F) Expression of GFAP in the cultured primary mouse astrocytes. DAPI (blue). (EPS 15343 kb)
Additional file 12: Figure S12. Sleep deprivation reduces the dextran radial distribution and ^125^I-apoE inflow from CSF into brain. A-B) Representative images of cascade blue dextran (CB) in mice on normal sleep cycle (A) and in mice during sleep deprivation (SD) (B). Cascade blue dextran (10 kDa) was injected into cisterna magna and the mice perfusion fixed (PFA) at 15 min. The vasculature was outline by lectin (green). Scale bars 100 μm (A-B). C) ^125^I-ApoE2 (yellow column), ^125^I-apoE3 (red column) and ^125^I-apoE4 (orange column) inflow into brain from the CSF were reduced in SD mice. D) ^14^C-inulin inflow into brain from the CSF was reduced with SD and not affected by apoE isoforms. ^125^I-ApoE (10 nM) and ^14^C-inulin were intracisternally injected and the brain analyzed for radioactivity. Values are mean ± SEM. *N* = 6 mice per group. (EPS 15099 kb)

## Abbreviations

ABCA1: ATP-binding cassette transporter A1
AD: Alzheimer’s disease
ANOVA: Analysis of variance
ApoA1: Apolipoprotein A1
ApoE: Apolipoprotein E
AQP4: Aquaporin 4
BBB: Blood–brain barrier
CAA: Cerebral amyloid angiopathy
CB: Cascade blue
CMV: Cytomegalovirus
CNS: Central nervous system
CPPT: Central polypurine tract (cPPT)
CSF: Cerebrospinal fluid
DAPI: 4',6-diamidino-2-phenylindole
DMEM: Dulbecco’s modified Eagle’s medium
DTT: Dithiothreitol
EGFP: Enhanced green fluorescent protein
ELISA: Enzyme-linked immunosorbent assay
FBS: Fetal bovine serum
FITC: Fluorescein isothiocyanate
FPLC: Fast protein liquid chromatography
GFAP: Glial fibrillary acidic protein
HBSS: Hank’s balanced salt solution
IRES: Internal ribosome entry site
ISF: Interstitial fluid
LDLR: Low-density lipoprotein receptor
Lenti: Lentivirus
LRP1: LDLR-related protein 1
LXR: Liver X receptors
ME: β-Mercaptoethanol
NeuN: Neuronal nuclear biomarker
NG2-DsRed: Red fluorescent protein variant (DsRed.T1) under the control of the mouse NG2 (*Cspg4*) promoter/enhancer)
PFA: Paraformaldehyde
TCA: Trichloroacetic acid
ZT: Zeitgeber time

## References

[1] Mahley RW, Rall SC (2000). Apolipoprotein E: far more than a lipid transport protein. Annu Rev Genomics Hum Genet.

[2] Linton MF, Gish R, Hubl ST, Butler E, Esquivel C, Bry WI, Boyles JK, Wardell MR, Young SG (1991). Phenotypes of Apolipoprotein-B and Apolipoprotein-E after Liver-Transplantation. J Clin Investig.

[3] Pitas RE, Boyles JK, Lee SH, Hui D, Weisgraber KH (1987). Lipoproteins and their receptors in the central nervous system. Characterization of the lipoproteins in cerebrospinal fluid and identification of apolipoprotein B, E(LDL) receptors in the brain. J Biol Chem.

[4] Hatters DM, Zhong N, Rutenber E, Weisgraber KH (2006). Amino-terminal domain stability mediates apolipoprotein E aggregation into neurotoxic fibrils. J Mol Biol.

[5] Verghese PB, Castellano JM, Holtzman DM (2011). Apolipoprotein E in Alzheimer’s disease and other neurological disorders. Lancet Neurol.

[6] Deane R, Sagare A, Hamm K, Parisi M, Lane S, Finn MB, Holtzman DM, Zlokovic BV (2008). apoE isoform-specific disruption of amyloid beta peptide clearance from mouse brain. J Clin Invest.

[7] Holtzman DM, Herz J, Bu G (2012). Apolipoprotein E and apolipoprotein E receptors: normal biology and roles in Alzheimer disease. Cold Spring Harb Perspect Med.

[8] Liu M, Kuhel DG, Shen L, Hui DY, Woods SC (2012). Apolipoprotein E does not cross the blood-cerebrospinal fluid barrier, as revealed by an improved technique for sampling CSF from mice. Am J Physiol Regul Integr Comp Physiol.

[9] Pfrieger FW, Ungerer N (2011). Cholesterol metabolism in neurons and astrocytes. Prog Lipid Res.

[10] Pfrieger FW (2003). Outsourcing in the brain: do neurons depend on cholesterol delivery by astrocytes?. BioEssays.

[11] Wahrle SE, Jiang H, Parsadanian M, Legleiter J, Han X, Fryer JD, Kowalewski T, Holtzman DM (2004). ABCA1 is required for normal central nervous system ApoE levels and for lipidation of astrocyte-secreted apoE. J Biol Chem.

[12] DeMattos RB, Brendza RP, Heuser JE, Kierson M, Cirrito JR, Fryer J, Sullivan PM, Fagan AM, Han X, Holtzman DM (2001). Purification and characterization of astrocyte-secreted apolipoprotein E and J-containing lipoproteins from wild-type and human apoE transgenic mice. Neurochem Int.

[13] Sun Y, Wu S, Bu G, Onifade MK, Patel SN, LaDu MJ, Fagan AM, Holtzman DM (1998). Glial fibrillary acidic protein-apolipoprotein E (apoE) transgenic mice: astrocyte-specific expression and differing biological effects of astrocyte-secreted apoE3 and apoE4 lipoproteins. J Neurosci.

[14] Fagan AM, Holtzman DM, Munson G, Mathur T, Schneider D, Chang LK, Getz GS, Reardon CA, Lukens J, Shah JA, LaDu MJ (1999). Unique lipoproteins secreted by primary astrocytes from wild type, apoE (−/−), and human apoE transgenic mice. J Biol Chem.

[15] Morikawa M, Fryer JD, Sullivan PM, Christopher EA, Wahrle SE, DeMattos RB, O’Dell MA, Fagan AM, Lashuel HA, Walz T (2005). Production and characterization of astrocyte-derived human apolipoprotein E isoforms from immortalized astrocytes and their interactions with amyloid-beta. Neurobiol Dis.

[16] Liu CC, Kanekiyo T, Xu H, Bu G (2013). Apolipoprotein E and Alzheimer disease: risk, mechanisms and therapy. Nat Rev Neurol.

[17] Xu Q, Bernardo A, Walker D, Kanegawa T, Mahley RW, Huang Y (2006). Profile and regulation of apolipoprotein E (ApoE) expression in the CNS in mice with targeting of green fluorescent protein gene to the ApoE locus. J Neurosci.

[18] Fryer JD, Demattos RB, McCormick LM, O’Dell MA, Spinner ML, Bales KR, Paul SM, Sullivan PM, Parsadanian M, Bu G, Holtzman DM (2005). The low density lipoprotein receptor regulates the level of central nervous system human and murine apolipoprotein E but does not modify amyloid plaque pathology in PDAPP mice. J Biol Chem.

[19] Ulrich JD, Burchett JM, Restivo JL, Schuler DR, Verghese PB, Mahan TE, Landreth GE, Castellano JM, Jiang H, Cirrito JR, Holtzman DM (2013). In vivo measurement of apolipoprotein E from the brain interstitial fluid using microdialysis. Mol Neurodegener.

[20] Fujiyoshi M, Ohtsuki S, Hori S, Tachikawa M, Terasaki T (2007). 24S-hydroxycholesterol induces cholesterol release from choroid plexus epithelial cells in an apical- and apoE isoform-dependent manner concomitantly with the induction of ABCA1 and ABCG1 expression. J Neurochem.

[21] Suon S, Zhao J, Villarreal SA, Anumula N, Liu M, Carangia LM, Renger JJ, Zerbinatti CV (2010). Systemic treatment with liver X receptor agonists raises apolipoprotein E, cholesterol, and amyloid-beta peptides in the cerebral spinal fluid of rats. Mol Neurodegener.

[22] Walker LC, Parker CA, Lipinski WJ, Callahan MJ, Carroll RT, Gandy SE, Smith JD, Jucker M, Bisgaier CL (1997). Cerebral lipid deposition in aged apolipoprotein-E-deficient mice. Am J Pathol.

[23] Sullivan PM, Han B, Liu F, Mace BE, Ervin JF, Wu S, Koger D, Paul S, Bales KR (2011). Reduced levels of human apoE4 protein in an animal model of cognitive impairment. Neurobiol Aging.

[24] Riddell DR, Zhou H, Atchison K, Warwick HK, Atkinson PJ, Jefferson J, Xu L, Aschmies S, Kirksey Y, Hu Y (2008). Impact of apolipoprotein E (ApoE) polymorphism on brain ApoE levels. J Neurosci.

[25] Mahley RW (1988). Apolipoprotein E: cholesterol transport protein with expanding role in cell biology. Science.

[26] Iliff JJ, Wang M, Liao Y, Plogg BA, Peng W, Gundersen GA, Benveniste H, Vates GE, Deane R, Goldman SA (2012). A paravascular pathway facilitates CSF flow through the brain parenchyma and the clearance of interstitial solutes, including amyloid beta. Sci Transl Med.

[27] Xie L, Kang H, Xu Q, Chen MJ, Liao Y, Thiyagarajan M, O’Donnell J, Christensen DJ, Nicholson C, Iliff JJ (2013). Sleep drives metabolite clearance from the adult brain. Science.

[28] Yang L, Kress BT, Weber HJ, Thiyagarajan M, Wang B, Deane R, Benveniste H, Iliff JJ, Nedergaard M (2013). Evaluating glymphatic pathway function utilizing clinically relevant intrathecal infusion of CSF tracer. J Transl Med.

[29] Rangroo Thrane V, Thrane AS, Plog BA, Thiyagarajan M, Iliff JJ, Deane R, Nagelhus EA, Nedergaard M (2013). Paravascular microcirculation facilitates rapid lipid transport and astrocyte signaling in the brain. Sci Rep.

[30] Peng W, Achariyar TM, Li B, Liao Y, Mestre H, Hitomi E, Regan S, Kasper T, Peng S, Ding F (2016). Suppression of glymphatic fluid transport in a mouse model of Alzheimer’s disease. Neurobiol Dis.

[31] Zhu X, Bergles DE, Nishiyama A (2008). NG2 cells generate both oligodendrocytes and gray matter astrocytes. Development.

[32] Thrane AS, Rappold PM, Fujita T, Torres A, Bekar LK, Takano T, Peng W, Wang F, Rangroo Thrane V, Enger R (2011). Critical role of aquaporin-4 (AQP4) in astrocytic Ca2+ signaling events elicited by cerebral edema. Proc Natl Acad Sci U S A.

[33] Zufferey R, Donello JE, Trono D, Hope TJ (1999). Woodchuck hepatitis virus posttranscriptional regulatory element enhances expression of transgenes delivered by retroviral vectors. J Virol.

[34] Klages N, Zufferey R, Trono D (2000). A stable system for the high-titer production of multiply attenuated lentiviral vectors. Mol Ther.

[35] Verghese PB, Castellano JM, Garai K, Wang Y, Jiang H, Shah A, Bu G, Frieden C, Holtzman DM (2013). ApoE influences amyloid-beta (Abeta) clearance despite minimal apoE/Abeta association in physiological conditions. Proc Natl Acad Sci U S A.

[36] LaDu MJ, Gilligan SM, Lukens JR, Cabana VG, Reardon CA, Van Eldik LJ, Holtzman DM (1998). Nascent astrocyte particles differ from lipoproteins in CSF. J Neurochem.

[37] Stukas S, Freeman L, Lee M, Wilkinson A, Ossoli A, Vaisman B, Demosky S, Chan J, Hirsch-Reinshagen V, Remaley AT, Wellington CL (2014). LCAT deficiency does not impair amyloid metabolism in APP/PS1 mice. J Lipid Res.

[38] Brightman MW, Reese TS (1969). Junctions between intimately apposed cell membranes in the vertebrate brain. J Cell Biol.

[39] Mathiisen TM, Lehre KP, Danbolt NC, Ottersen OP (2010). The perivascular astroglial sheath provides a complete covering of the brain microvessels: an electron microscopic 3D reconstruction. Glia.

[40] Rangroo Thrane V, Thrane AS, Wang F, Cotrina ML, Smith NA, Chen M, Xu Q, Kang N, Fujita T, Nagelhus EA, Nedergaard M (2013). Ammonia triggers neuronal disinhibition and seizures by impairing astrocyte potassium buffering. Nat Med.

[41] Wang M, Iliff JJ, Liao Y, Chen MJ, Shinseki MS, Venkataraman A, Cheung J, Wang W, Nedergaard M (2012). Cognitive deficits and delayed neuronal loss in a mouse model of multiple microinfarcts. J Neurosci.

[42] Kress BT, Iliff JJ, Xia M, Wang M, Wei HS, Zeppenfeld D, Xie L, Kang H, Xu Q, Liew JA, et al.. Impairment of paravascular clearance pathways in the aging brain. Ann Neurol. 2014;76(6):845–61. 10.1002/ana.24271 PMC4245362 25204284

[43] Zhang J, Zhu Y, Zhan G, Fenik P, Panossian L, Wang MM, Reid S, Lai D, Davis JG, Baur JA, Veasey S (2014). Extended wakefulness: compromised metabolics in and degeneration of locus ceruleus neurons. J Neurosci.

[44] Bell RD, Sagare AP, Friedman AE, Bedi GS, Holtzman DM, Deane R, Zlokovic BV (2007). Transport pathways for clearance of human Alzheimer’s amyloid beta-peptide and apolipoproteins E and J in the mouse central nervous system. J Cereb Blood Flow Metab.

[45] Deane R, Wu Z, Sagare A, Davis J, Du Yan S, Hamm K, Xu F, Parisi M, LaRue B, Hu HW (2004). LRP/amyloid beta-peptide interaction mediates differential brain efflux of Abeta isoforms. Neuron.

[46] Plog BA, Dashnaw ML, Hitomi E, Peng W, Liao Y, Lou N, Deane R, Nedergaard M (2015). Biomarkers of traumatic injury are transported from brain to blood via the glymphatic system. J Neurosci.

[47] Fabis MJ, Phares TW, Kean RB, Koprowski H, Hooper DC (2008). Blood–brain barrier changes and cell invasion differ between therapeutic immune clearance of neurotrophic virus and CNS autoimmunity. Proc Natl Acad Sci U S A.

[48] Monnot AD, Zheng W (2013). Culture of choroid plexus epithelial cells and in vitro model of blood-CSF barrier. Methods Mol Biol.

[49] Hudry E, Dashkoff J, Roe AD, Takeda S, Koffie RM, Hashimoto T, Scheel M, Spires-Jones T, Arbel-Ornath M, Betensky R (2013). Gene transfer of human Apoe isoforms results in differential modulation of amyloid deposition and neurotoxicity in mouse brain. Sci Transl Med.

[50] Thal DR, Larionov S, Abramowski D, Wiederhold KH, Van Dooren T, Yamaguchi H, Haass C, Van Leuven F, Staufenbiel M, Capetillo-Zarate E (2007). Occurrence and co-localization of amyloid beta-protein and apolipoprotein E in perivascular drainage channels of wild-type and APP-transgenic mice. Neurobiol Aging.

[51] Rolyan H, Feike AC, Upadhaya AR, Waha A, Van Dooren T, Haass C, Birkenmeier G, Pietrzik CU, Van Leuven F, Thal DR (2011). Amyloid-beta protein modulates the perivascular clearance of neuronal apolipoprotein E in mouse models of Alzheimer’s disease. J Neural Transm.

[52] McCaslin AF, Chen BR, Radosevich AJ, Cauli B, Hillman EM (2011). In vivo 3D morphology of astrocyte-vasculature interactions in the somatosensory cortex: implications for neurovascular coupling. J Cereb Blood Flow Metab.

[53] Innerarity TL, Pitas RE, Mahley RW (1979). Binding of arginine-rich (E) apoprotein after recombination with phospholipid vesicles to the low density lipoprotein receptors of fibroblasts. J Biol Chem.

[54] Kowal RC, Herz J, Weisgraber KH, Mahley RW, Brown MS, Goldstein JL (1990). Opposing effects of apolipoproteins E and C on lipoprotein binding to low density lipoprotein receptor-related protein. J Biol Chem.

[55] Innerarity TL, Pitas RE, Mahley RW (1986). Lipoprotein-receptor interactions. Methods Enzymol.

[56] Kim WS, Elliott DA, Kockx M, Kritharides L, Rye KA, Jans DA, Garner B (2008). Analysis of apolipoprotein E nuclear localization using green fluorescent protein and biotinylation approaches. Biochem J.

[57] Theendakara V, Peters-Libeu CA, Spilman P, Poksay KS, Bredesen DE, Rao RV (2016). Direct transcriptional effects of apolipoprotein E. J Neurosci.

[58] Liu G, Martins IH, Chiorini JA, Davidson BL (2005). Adeno-associated virus type 4 (AAV4) targets ependyma and astrocytes in the subventricular zone and RMS. Gene Ther.

[59] Yang G, Lai CS, Cichon J, Ma L, Li W, Gan WB (2014). Sleep promotes branch-specific formation of dendritic spines after learning. Science.

[60] Jackson ML, Gunzelmann G, Whitney P, Hinson JM, Belenky G, Rabat A, Van Dongen HP (2013). Deconstructing and reconstructing cognitive performance in sleep deprivation. Sleep Med Rev.

[61] Scullin MK, Bliwise DL (2015). Is cognitive aging associated with levels of REM sleep or slow wave sleep?. Sleep.

[62] Musiek ES (2015). Circadian clock disruption in neurodegenerative diseases: cause and effect?. Front Pharmacol.

[63] Davson H, Segal MB (1970). The effects of some inhibitors and accelerators of sodium transport on the turnover of 22Na in the cerebrospinal fluid and the brain. J Physiol.

[64] Johanson CE, Duncan JA, Klinge PM, Brinker T, Stopa EG, Silverberg GD (2008). Multiplicity of cerebrospinal fluid functions: New challenges in health and disease. Cerebrospinal Fluid Res.

[65] Pollay M (2010). The function and structure of the cerebrospinal fluid outflow system. Cerebrospinal Fluid Res.

[66] Dickson PW, Aldred AR, Menting JG, Marley PD, Sawyer WH, Schreiber G (1987). Thyroxine transport in choroid plexus. J Biol Chem.

[67] Kassem NA, Deane R, Segal MB, Preston JE (2006). Role of transthyretin in thyroxine transfer from cerebrospinal fluid to brain and choroid plexus. Am J Physiol Regul Integr Comp Physiol.

[68] Stukas S, Robert J, Lee M, Kulic I, Carr M, Tourigny K, Fan J, Namjoshi D, Lemke K, DeValle N (2014). Intravenously injected human apolipoprotein A-I rapidly enters the central nervous system via the choroid plexus. J Am Heart Assoc.

[69] Hirsch-Reinshagen V, Zhou S, Burgess BL, Bernier L, McIsaac SA, Chan JY, Tansley GH, Cohn JS, Hayden MR, Wellington CL (2004). Deficiency of ABCA1 impairs apolipoprotein E metabolism in brain. J Biol Chem.

[70] Xie C, Burns DK, Turley SD, Dietschy JM (2000). Cholesterol is sequestered in the brains of mice with Niemann-Pick type C disease but turnover is increased. J Neuropathol Exp Neurol.

[71] Reid PC, Sakashita N, Sugii S, Ohno-Iwashita Y, Shimada Y, Hickey WF, Chang TY (2004). A novel cholesterol stain reveals early neuronal cholesterol accumulation in the Niemann-Pick type C1 mouse brain. J Lipid Res.

[72] Huang Y, Weisgraber KH, Mucke L, Mahley RW (2004). Apolipoprotein E: diversity of cellular origins, structural and biophysical properties, and effects in Alzheimer’s disease. J Mol Neurosci.

[73] Iliff JJ, Chen MJ, Plog BA, Zeppenfeld DM, Soltero M, Yang L, Singh I, Deane R, Nedergaard M (2014). Impairment of glymphatic pathway function promotes tau pathology after traumatic brain injury. J Neurosci.

[74] Cserr HF, Ostrach LH (1974). Bulk flow of interstitial fluid after intracranial injection of blue dextran 2000. Exp Neurol.

[75] Cserr HF, Cooper DN, Milhorat TH (1977). Flow of cerebral interstitial fluid as indicated by the removal of extracellular markers from rat caudate nucleus. Exp Eye Res.

[76] Szentistvanyi I, Patlak CS, Ellis RA, Cserr HF (1984). Drainage of interstitial fluid from different regions of rat brain. Am J Physiol.

[77] Cserr HF (1988). Role of secretion and bulk flow of brain interstitial fluid in brain volume regulation. Ann N Y Acad Sci.

[78] Brinker T, Stopa E, Morrison J, Klinge P (2014). A new look at cerebrospinal fluid circulation. Fluids Barriers CNS.

[79] Marchi N, Banjara M, Janigro D (2016). Blood–brain barrier, bulk flow, and interstitial clearance in epilepsy. J Neurosci Methods.

[80] Rosenberg GA, Kyner WT, Estrada E (1980). Bulk flow of brain interstitial fluid under normal and hyperosmolar conditions. Am J Physiol.

[81] Bakker EN, Bacskai BJ, Arbel-Ornath M, Aldea R, Bedussi B, Morris AW, Weller RO, Carare RO (2016). Lymphatic clearance of the brain: perivascular, paravascular and significance for neurodegenerative diseases. Cell Mol Neurobiol.

[82] Stokum JA, Gerzanich V, Simard JM (2016). Molecular pathophysiology of cerebral edema. J Cereb Blood Flow Metab.

[83] Bedussi B, van Lier MG, Bartstra JW, de Vos J, Siebes M, VanBavel E, Bakker EN (2015). Clearance from the mouse brain by convection of interstitial fluid towards the ventricular system. Fluids Barriers CNS.

[84] Bedussi B, van der Wel NN, de Vos J, van Veen H, Siebes M, VanBavel E, Bakker EN. Paravascular channels, cisterns, and the subarachnoid space in the rat brain: A single compartment with preferential pathways. J Cereb Blood Flow Metab. 2016. Epub ahead of print. PMID27306753. 10.1177/0271678X16655550 PMC5453458 27306753

[85] Weller RO, Galea I, Carare RO, Minagar A (2010). Pathophysiology of the lymphatic drainage of the central nervous system: Implications for pathogenesis and therapy of multiple sclerosis. Pathophysiology.

[86] Morris AW, Sharp MM, Albargothy NJ, Fernandes R, Hawkes CA, Verma A, Weller RO, Carare RO (2016). Vascular basement membranes as pathways for the passage of fluid into and out of the brain. Acta Neuropathol.

[87] Weller RO, Kida S, Zhang ET (1992). Pathways of fluid drainage from the brain—morphological aspects and immunological significance in rat and man. Brain Pathol.

[88] Hawkes CA, Hartig W, Kacza J, Schliebs R, Weller RO, Nicoll JA, Carare RO (2011). Perivascular drainage of solutes is impaired in the ageing mouse brain and in the presence of cerebral amyloid angiopathy. Acta Neuropathol.

[89] Harris FM, Tesseur I, Brecht WJ, Xu Q, Mullendorff K, Chang S, Wyss-Coray T, Mahley RW, Huang Y (2004). Astroglial regulation of apolipoprotein E expression in neuronal cells. Implications for Alzheimer’s disease. J Biol Chem.

[90] Wildsmith KR, Basak JM, Patterson BW, Pyatkivskyy Y, Kim J, Yarasheski KE, Wang JX, Mawuenyega KG, Jiang H, Parsadanian M (2012). In vivo human apolipoprotein E isoform fractional turnover rates in the CNS. PLoS One.

[91] Kanekiyo T, Liu CC, Shinohara M, Li J, Bu G (2012). LRP1 in brain vascular smooth muscle cells mediates local clearance of Alzheimer’s amyloid-beta. J Neurosci.

[92] Bu G (2009). Apolipoprotein E and its receptors in Alzheimer’s disease: pathways, pathogenesis and therapy. Nat Rev Neurosci.

[93] Herz J (2009). Apolipoprotein E receptors in the nervous system. Curr Opin Lipidol.

[94] Ruiz J, Kouiavskaia D, Migliorini M, Robinson S, Saenko EL, Gorlatova N, Li D, Lawrence D, Hyman BT, Weisgraber KH, Strickland DK (2005). The apoE isoform binding properties of the VLDL receptor reveal marked differences from LRP and the LDL receptor. J Lipid Res.

[95] Narita M, Holtzman DM, Fagan AM, LaDu MJ, Yu L, Han X, Gross RW, Bu G, Schwartz AL (2002). Cellular catabolism of lipid poor apolipoprotein E via cell surface LDL receptor-related protein. J Biochem.

[96] Weisgraber KH, Rall SC, Mahley RW (1981). Human E apoprotein heterogeneity. Cysteine-arginine interchanges in the amino acid sequence of the apo-E isoforms. J Biol Chem.

[97] Acosta-Peña E, Camacho-Abrego I, Melgarejo-GutiErrez M, Flores G, Drucker-ColIn R, GarcIa-GarcIa F. Sleep deprivation induces differential morphological changes in the hippocampus and prefrontal cortex in young and old rats. Synapse. 2015;69(1):15–25. 10.1002/syn.21779 25179486

[98] Longordo F, Kopp C, Luthi A (2009). Consequences of sleep deprivation on neurotransmitter receptor expression and function. Eur J Neurosci.

[99] Hipolide DC, Moreira KM, Barlow KB, Wilson AA, Nobrega JN, Tufik S (2005). Distinct effects of sleep deprivation on binding to norepinephrine and serotonin transporters in rat brain. Prog Neuropsychopharmacol Biol Psychiatry.

[100] Irwin M, Thompson J, Miller C, Gillin JC, Ziegler M (1999). Effects of sleep and sleep deprivation on catecholamine and interleukin-2 levels in humans: clinical implications. J Clin Endocrinol Metab.

[101] Kalonia H, Bishnoi M, Kumar A (2008). Possible mechanism involved in sleep deprivation-induced memory dysfunction. Methods Find Exp Clin Pharmacol.

[102] Venkatraman V, Huettel SA, Chuah LY, Payne JW, Chee MW (2011). Sleep deprivation biases the neural mechanisms underlying economic preferences. J Neurosci.

[103] Kato M, Phillips BG, Sigurdsson G, Narkiewicz K, Pesek CA, Somers VK (2000). Effects of sleep deprivation on neural circulatory control. Hypertension.

[104] Cipolla MJ, Li R, Vitullo L (2004). Perivascular innervation of penetrating brain parenchymal arterioles. J Cardiovasc Pharmacol.

[105] Zhu Y, Nwabuisi-Heath E, Dumanis SB, Tai LM, Yu C, Rebeck GW, LaDu MJ (2012). APOE genotype alters glial activation and loss of synaptic markers in mice. Glia.

[106] Dumanis SB, Cha HJ, Song JM, Trotter JH, Spitzer M, Lee JY, Weeber EJ, Turner RS, Pak DT, Rebeck GW, Hoe HS (2011). ApoE receptor 2 regulates synapse and dendritic spine formation. PLoS One.

[107] Dumanis SB, Tesoriero JA, Babus LW, Nguyen MT, Trotter JH, Ladu MJ, Weeber EJ, Turner RS, Xu B, Rebeck GW, Hoe HS (2009). ApoE4 decreases spine density and dendritic complexity in cortical neurons in vivo. J Neurosci.

[108] Korwek KM, Trotter JH, Ladu MJ, Sullivan PM, Weeber EJ (2009). ApoE isoform-dependent changes in hippocampal synaptic function. Mol Neurodegener.

[109] Hawkes CA, Sullivan PM, Hands S, Weller RO, Nicoll JA, Carare RO (2012). Disruption of arterial perivascular drainage of amyloid-beta from the brains of mice expressing the human APOE epsilon4 allele. PLoS One.

[110] Premkumar DRD, Cohen DL, Hedera P, Friedland RP, Kalaria RN (1996). Apolipoprotein E-epsilon 4 alleles in cerebral amyloid angiopathy and cerebrovascular pathology associated with Alzheimer’s disease. Am J Pathol.

[111] McCarron MO, Nicoll JA, Ironside JW, Love S, Alberts MJ, Bone I (1999). Cerebral amyloid angiopathy-related hemorrhage. Interaction of APOE epsilon2 with putative clinical risk factors. Stroke.

[112] Greenberg SM, Vonsattel JP, Segal AZ, Chiu RI, Clatworthy AE, Liao A, Hyman BT, Rebeck GW (1998). Association of apolipoprotein E epsilon2 and vasculopathy in cerebral amyloid angiopathy. Neurology.

[113] Greenberg SM, Briggs ME, Hyman BT, Kokoris GJ, Takis C, Kanter DS, Kase CS, Pessin MS (1996). Apolipoprotein E epsilon 4 is associated with the presence and earlier onset of hemorrhage in cerebral amyloid angiopathy. Stroke.

[114] Hu J, Liu C-C, Chen X-F, Zhang Y-w, Xu H, Bu G (2015). Opposing effects of viral mediated brain expression of apolipoprotein E2 (apoE2) and apoE4 on apoE lipidation and Abeta metabolism in apoE4-targeted replacement mice. Mol Neurodegener.

[115] Dodart JC, Marr RA, Koistinaho M, Gregersen BM, Malkani S, Verma IM, Paul SM (2005). Gene delivery of human apolipoprotein E alters brain Abeta burden in a mouse model of Alzheimer’s disease. Proc Natl Acad Sci U S A.

[116] Bien-Ly N, Gillespie AK, Walker D, Yoon SY, Huang Y (2012). Reducing human apolipoprotein E levels attenuates age-dependent Abeta accumulation in mutant human amyloid precursor protein transgenic mice. J Neurosci.

[117] Lane-Donovan C, Herz J (2014). Is apolipoprotein e required for cognitive function in humans?: implications for Alzheimer drug development. JAMA Neurol.

