# Erratum to: Glymphatic distribution of CSF-derived apoE into brain is isoform specific and suppressed during sleep deprivation

**DOI:** 10.1186/s13024-016-0147-7

**Published:** 2017-01-12

**Authors:** Thiyagaragan M. Achariyar, Baoman Li, Weiguo Peng, Philip B. Verghese, Yang Shi, Evan McConnell, Abdellatif Benraiss, Tristan Kasper, Wei Song, Takahiro Takano, David M. Holtzman, Maiken Nedergaard, Rashid Deane

**Affiliations:** 1 Center for Translational Neuromedicine, Division of Glial Disease and Therapeutics, Department of Neurosurgery, University of Rochester Medical Center, University of Rochester, Rochester, NY 14642 USA; 2 Department of Neurology, Hope Center for Neurological Disorders, and the Charles F. and Joanne Knight Alzheimer’s Disease Research Center, Washington University School of Medicine, St Louis, MO 63110 USA; 3 Center for Translational Neuromedicine, Division of Cell and Gene Therapy, University of Rochester Medical Center, Rochester, NY 14642 USA; 4 Laboratory of Brain Metabolic Diseases, Institute of Metabolic Disease Research and Drug Development, China Medical University, Shenyang, China

## Erratum

The original article [1] contains an error whereby the co-author, Takahiro Takano’s name was misspelt.

This error has now been corrected in the original article.
